# A phosphorylation-driven ubiquitination switch fine-tunes Alfin-like 7–induced ROS signaling in plant immunity

**DOI:** 10.1126/sciadv.adw7554

**Published:** 2025-06-13

**Authors:** Dingliang Zhang, Xinyu Zhang, Zhiyan Wen, Yiping Wang, Lizhi Tan, Zhaolei Li, Wenli Li, Yi Li, Xiaofei Zhao, Meng Yang, Zhen Li, Dawei Li, Savithramma P. Dinesh-Kumar, Yongliang Zhang

**Affiliations:** ^1^ State Key Laboratory of Plant Environmental Resilience, College of Biological Sciences, China Agricultural University, Beijing 100193, China.; ^2^ Department of Plant Biology and The Genome Center, College of Biological Sciences, University of California, Davis, Davis, CA 95616, USA.

## Abstract

Maintaining reactive oxygen species (ROS) homeostasis is essential for balancing growth-defense trade-offs in plants. Although the transcription factors (TFs) that regulate ROS production and scavenging genes have been studied, the regulation of these TFs to control ROS accumulation remains poorly understood. Here, we demonstrate that during N nucleotide-binding leucine-rich repeat–mediated immunity, Alfin-like 7 (AL7) is ubiquitinated by the ubiquitin protein ligase E3 component N-recognin 7 (UBR7). UBR7 interacts with AL7 and acts as a molecular brake to mediate the ubiquitination of AL7 at lysine-20, leading to its subsequent proteasomal degradation. UBR7 functions upstream of AL7 to reduce AL7-induced ROS accumulation during *N*-mediated defense. The phosphorylation of AL7 at serine-174 enhances its interaction with UBR7, thereby increasing AL7 ubiquitination and reducing AL7 stability. Our findings reveal a mechanism by which ROS accumulation is regulated through the phosphorylation and ubiquitination of a TF during the immune response. This ensures precise switching of ROS signaling to prevent excessive defense responses.

## Teaser

Phosphorylation and ubiquitination of AL7 fine-tune ROS homeostasis at the transcriptional level during ETI responses.

## INTRODUCTION

Plants are often subjected to pathogen attacks due to their sessile lifestyle. To combat these infections, they have evolved a sophisticated immune system composed of two defense layers: pattern-triggered immunity (PTI) and effector-triggered immunity (ETI) (*1*). Pattern recognition receptors, located on the cell surface or intracellular immune receptors such as nucleotide-binding leucine-rich repeat (NLR) proteins, recognize pathogen-associated molecular patterns or pathogen effectors, respectively, to activate downstream immune signaling (*2*). These pathways typically involve bursts of reactive oxygen species (ROS), calcium (Ca^2+^) influx, and transcriptional reprogramming events (*3*–*10*).

ROS has dual roles in plants, acting both as toxic compounds and as key regulators of numerous biological processes, including plant immune responses (*3*, *11*). Maintaining ROS homeostasis is crucial for normal plant growth and defense responses to avoid fitness costs. ROS levels are determined by a tightly controlled balance between ROS production and breakdown, achieved through sophisticated and complex mechanisms (*11*, *12*). For example, reduced form of nicotinamide adenine dinucleotide phosphate (NADPH) oxidases, one of the major sources of ROS in plants, are frequently regulated by various posttranslational modifications to orchestrate ROS production (*13*). In addition, plants deploy antioxidant systems, such as peroxidases, ascorbate oxidases, and catalases, to scavenge excess ROS and maintain ROS homeostasis (*14*). For instance, the Ca^2+^ sensor resistance of rice to disease 1 (ROD1) has been reported to promote ROS scavenging by stimulating catalase activity (*15*). In addition to regulating the functions of NADPH oxidases and antioxidant systems at the protein level, various transcription factors target the promoters of respiratory burst oxidase homologues (RBOHs) and antioxidant genes to manage ROS accumulation at the transcriptional level (*9*, *16*). However, the exact mechanisms by which these transcription factors affect ROS production, including their activation and deactivation to restore homeostasis during plant immune responses, remain poorly understood.

We previously identified a transcriptional repressor, Alfin-like 7 (AL7), that promotes ROS accumulation by suppressing the expression of ROS-scavenging genes, thereby enhancing *N*-mediated resistance to tobacco mosaic virus (TMV) (*17*). However, it remains unclear how AL7’s function in promoting ROS is turned off to restore ROS homeostasis and prevent adverse effects on normal plant growth and development. In this study, we found that AL7 undergoes ubiquitination during *N*-mediated immune responses and a putative E3 ubiquitin ligase, UBR7, mediates AL7 ubiquitination and subsequent degradation via the 26*S* proteasome. Furthermore, the phosphorylation of AL7 increases its ubiquitination by UBR7, thereby reducing its stability and mitigating AL7-induced ROS accumulation. Our study reveals a phosphorylation-driven ubiquitination switch for the dynamic regulation of ROS homeostasis during plant immune responses.

## RESULTS

### Ubiquitination of AL7 during *N*-mediated immune response

**Fig. 1.**
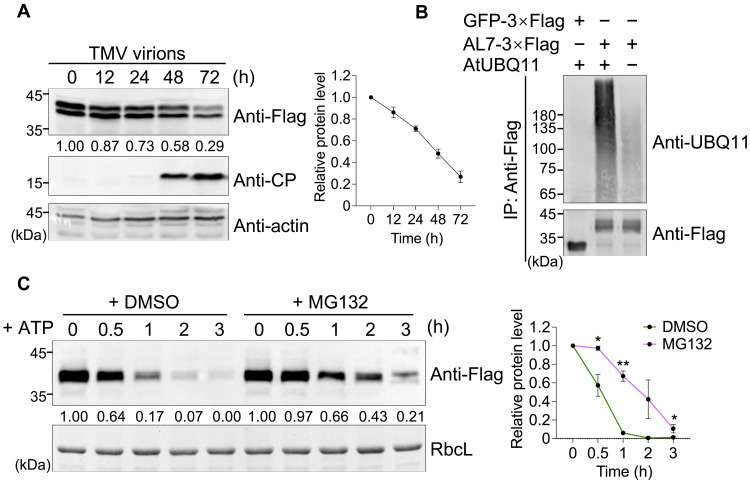
AL7 undergoes ubiquitination and is degraded by the 26*S* proteasome. (**A**) Immunoblot analysis of AL7 protein levels in *AL7-OE* transgenic plants during TMV infection. Total proteins were extracted from *AL7-OE* plants at the indicated time points following the rub inoculation of 200 ng of TMV-U1 virions. The antibodies used are shown on the right side of the panel. Actin served as the loading control. h, hours; CP, coat protein. (**B**) Immunoblot analysis of AL7 ubiquitination in vivo. Total proteins from infiltrated leaves were immunoprecipitated with anti-Flag beads and detected by Western blot using the antibodies indicated on the right. (**C**) Cell-free degradation assay to test AL7 stability. The leaf extracts were treated with 10 mM ATP, 0.5 mM cycloheximide (CHX), and 50 μM MG132 (+MG132) or an equal concentration of dimethyl sulfoxide (DMSO) (+DMSO) to assess the varying periods indicated above the panel. Immunoblot analysis using anti-Flag antibody was conducted to evaluate AL7 protein levels. RbcL served as the loading control. A quantitative analysis of the AL7 degradation rate is shown on the right. Error bars represent the means ± SD (*n* = 3 biological replicates). Asterisks above the corresponding line chart indicate significant differences between the MG132-treated and DMSO-treated control samples, as determined by an unpaired two-tailed *t* test (*P < 0.05; **P < 0.01). All experiments were conducted three times with similar results.

We previously reported that AL7 positively regulates the *N*-mediated immune response by suppressing the expression of ROS-scavenging genes and promoting ROS accumulation (*17*). Since excessive ROS production can be toxic to plants, they have evolved mechanisms to restore ROS homeostasis (*13*). To investigate how plants fine-tune AL7’s promotion of ROS production, we first analyzed changes in AL7 protein accumulation during *N*-mediated immunity using *N*-containing *Nicotiana benthamiana* (*NN*) plants that stably overexpress AL7 (*AL7-OE*). Immunoblot analysis revealed that AL7 protein accumulation gradually decreased as TMV infection progressed (Fig. 1A). Moreover, AL7 was coexpressed with either an empty vector (EV) or TMV–green fluorescent protein (GFP) in nontransgenic (NT) and *NN* plants. The results showed that TMV infection reduced AL7 protein levels in *NN* plants (fig. S1A), consistent with the findings presented in Fig. 1A. In contrast, no degradation of AL7 protein was observed in GFP-tagged TMV (TMV-GFP)–infected NT plants (fig. S1B). These results indicate that TMV infection induces AL7 degradation in an *N* NLR-dependent manner.

To further examine whether the reduction in AL7 protein accumulation is associated with ubiquitination, we coexpressed GFP-3×Flag or AL7-3×Flag with *Arabidopsis* UBIQUITIN 11 (AtUBQ11) in *N. benthamiana* leaves through agroinfiltration. At 38 hours postinfiltration (hpi), the leaves were treated with 100 μM of the 26*S* proteasome inhibitor MG132 and collected 10 hours later. Immunoblot analysis using an anti-UBQ11 antibody revealed that the AL7 protein underwent ubiquitination, while the GFP control protein did not (Fig. 1B). A cell-free degradation assay further demonstrated that the AL7 protein was progressively degraded in the presence of adenosine 5ʹ-triphosphate (ATP), whereas treatment with MG132 significantly reduced AL7 degradation (Fig. 1C). These results indicate that AL7 undergoes ubiquitin-mediated degradation during the *N*-mediated immune response.

### Proxitome profiling reveals the AL7 interaction network

To identify the E3 ubiquitin ligase responsible for the degradation of AL7 during *N*-mediated immune responses, we used a TurboID-based proximity labeling (PL) approach (*18*) to analyze AL7’s proxitome in both resting and immune-activated states. As a control, we fused a well-characterized nuclear localization signal (NLS) PKKKRKV from the SV40 large T antigen to direct the citrine-TurboID fusion to the nucleus (fig. S2, A and B). We initially transiently expressed the AL7-TurboID fusion in Cas9-free *AL7-KO* plants; the results indicated that it successfully rescued *N*-mediated resistance against TMV, demonstrating that the TurboID fusion at the C terminus of AL7 has no apparent effect on its function (fig. S2, C and D).

We expressed AL7-TurboID or NLS-citrine-TurboID in *NN* plants, with the presence (+p50) or absence (−p50) of the TMV p50 effector (fig. S2E). Immunoblot analysis confirmed the expression and biotinylation of both AL7-TurboID and NLS-citrine-TurboID. Biotinylated proteins were efficiently enriched using streptavidin beads (fig. S1, F and G). After on-bead digestion, the enriched proteins were analyzed through liquid chromatography–tandem mass spectrometry (LC-MS/MS) (fig. S2E).

A total of 5800 and 5639 proteins were identified in the AL7-TurboID samples under –p50 and +p50 conditions, respectively (data S1). Among these, 867 and 973 proteins were enriched interactors based on thresholds of *P* < 0.05 and fold change (AL7/NLS-citrine) ≥ 1.5 (fig. S3, A and B, and data S1). Hierarchical clustering and scatterplots demonstrated clear separation between the AL7-TurboID and control citrine-TurboID groups (fig. S3, C and D, and data S2). By comparing the proximal proteins of AL7 under resting and immune-activated conditions, we identified 532 proteins interacting with AL7 in both states, 335 unique to the resting state, and 441 unique to the immune-activated state (fig. S3E and data S3).

A recent report indicates that the *Arabidopsis* AL7 (AtAL7) ortholog is associated with 1245 proteins through affinity purification–MS analysis (*19*). Comparing our PL data with theirs revealed 116 shared proteins, including experimentally validated interactors of AtAL7, such as AL1 and transcription factor GATAs, which also appeared in our MS dataset (fig. S3, A, B, and F, and data S4). In addition, predictions of subcellular localization suggest that ~56 and 53% of AL7-enriched interactors localize to the nucleus in the absence and presence of p50, respectively (fig. S3, G and H, and data S5), supporting AL7’s function as a transcription factor that resides in the nucleus. Collectively, these findings reinforce our strong confidence in the PL data.

To characterize the functions of AL7’s proximal proteins, we conducted an analysis using the Kyoto Encyclopedia of Genes and Genomes (KEGG). The results indicated that AL7’s proximal proteins are involved in cellular processes, metabolic processes, RNA processing, and responses to external stimuli or stresses. Several pathways related to plant immunity and defense responses were significantly enriched (fig. S3, I and J, and data S6). To explore the associations among AL7’s proximal proteins, we constructed interaction networks under resting and immune-activated conditions using STRING (https://cn.string-db.org) and GeneMANIA (www.genemania.org). The results revealed that many of AL7’s proximal proteins are associated with defense and immunity, consistent with the role of the AL7 transcription factor in the *N*-mediated immune response (*17*). In addition, the interaction network suggested that AL7 may collaborate with other transcription factors to regulate transcriptional reprogramming events during *N*-mediated immunity (fig. S4, A and B). Furthermore, a subset of AL7’s proximal proteins is involved in ubiquitination processes (fig. S4, A and B), further supporting the idea that AL7 is subject to ubiquitination-mediated regulation in vivo.

### UBR7 interacts with AL7 in vivo and in vitro

When analyzing the PL data for AL7, UBR7, the mammalian homolog of a putative E3 ubiquitin ligase, frequently appeared and was enriched both in the absence and presence of p50 (fig. S3, A and B). Our previous PL-based analysis of the N NLR immune receptor complex identified UBR7 as a previously unidentified component of the N complex (*20*). However, the mechanism by which UBR7 modulates *N*-mediated immunity and whether it has E3 ubiquitin ligase activity to facilitate substrate degradation remains to be fully explored.

**Fig. 2.**
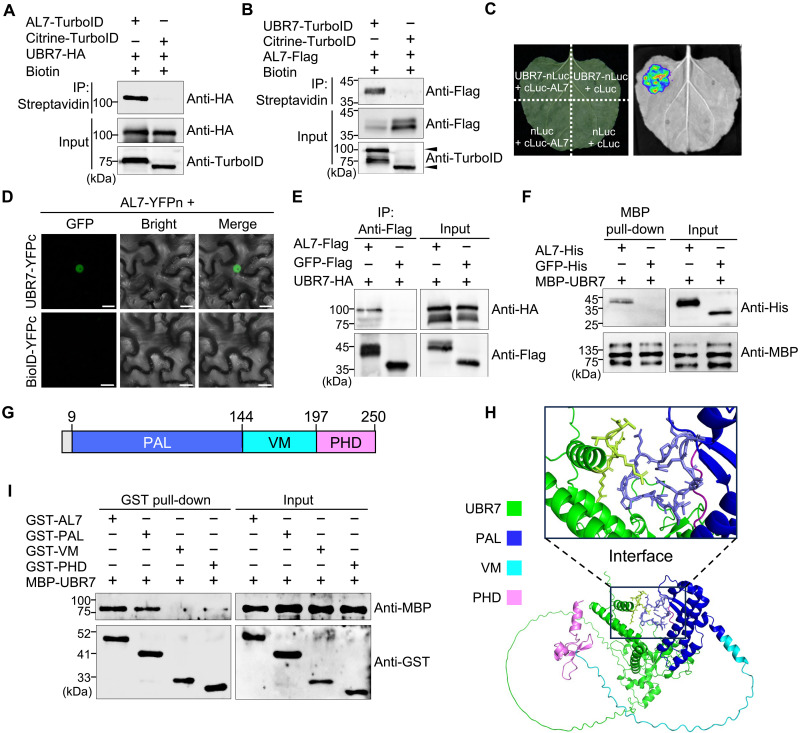
UBR7 interacts with AL7 in vivo and in vitro. (**A** and **B**) The interaction between AL7 and UBR7 was analyzed using TurboID-based PL in conjunction with streptavidin-based enrichment. UBR7-HA was coexpressed with either AL7-TurboID or the control NLS-citrine-TurboID (A), while AL7-Flag was coexpressed with UBR7-TurboID or the control citrine-TurboID in *N. benthamiana* leaves, followed by infiltration with 200 mM biotin at 40 hpi. Leaf tissues were harvested 8 hours later for subsequent enrichment using streptavidin beads and analysis by immunoblotting. (**C**) An LCI assay was conducted to analyze the interaction between UBR7 and AL7, with luminescence signals recorded at 48 hpi. cLuc, C terminal fragments of firefly luciferase; nLuc, N terminal fragments of firefly luciferase. (**D**) A BiFC assay was used to test the interaction between UBR7 and AL7. C-terminal fragments of yellow fluorescent protein (YFPc)-fused UBR7 (UBR7-YFPc) and control BioID-YFPc were coexpressed with N-terminal fragments of yellow fluorescent protein (YFPn)-fused AL7 (AL7-YFPn) in *N. benthamiana* leaves, respectively. Scale bars, 15 μm. (**E**) Co-IP analysis was performed to investigate the interaction between UBR7 and AL7. *N. benthamiana* leaves agroinfiltrated with the indicated combinations above the panel were harvested at 48 hpi, followed by immunoprecipitation (IP) using anti-Flag beads and subsequent immunoblot analysis with antibodies shown on the right side of the panels. (**F**) An MBP pull-down assay demonstrated the physical interaction between UBR7 and AL7. MBP-UBR7 was incubated with AL7-His or GFP-His. Input and pull-down products were analyzed by immunoblotting with antibodies indicated on the right side of the panels. (**G**) A schematic representation outlines the domain architecture of AL7. (**H**) Interaction interface predictions between UBR7 and AL7 were made using AlphaFold 3, with the domains of AL7 highlighted by different colored frames. (**I**) The MBP pull-down assay identified the domains of AL7 that interact with UBR7. All experiments were repeated at least twice with consistent results.

To validate the interaction between AL7 and UBR7, we coexpressed UBR7–hemagglutinin (HA) with AL7-TurboID and a control NLS-citrine-TurboID. Pull-down assays using streptavidin agarose, followed by immunoblotting with anti-HA antibodies, revealed the presence of UBR7 in the AL7-TurboID samples but not in the NLS-citrine-TurboID control (Fig. 2A). Similarly, immunoblotting with anti-Flag antibodies detected AL7 in the UBR7-TurboID samples but not in the citrine-TurboID control (Fig. 2B). In addition, luciferase complementation imaging (LCI), bimolecular fluorescence complementation (BiFC), and coimmunoprecipitation (Co-IP) assays confirmed the interaction between AL7 and UBR7, while the BioID or GFP-Flag controls did not show any interaction (Fig. 2, C to E). To determine whether UBR7 directly interacts with AL7, we performed maltodextrin binding protein (MBP) pull-down assays using purified MBP-tagged UBR7 (MBP-UBR7), His-tagged GFP (GFP-His), and His-tagged AL7 (AL7-His) from *Escherichia coli*. The results showed that AL7-His, but not GFP-His, was pulled down by MBP-UBR7, indicating a physical interaction between UBR7 and AL7 (Fig. 2F). To identify the domains responsible for the UBR7-AL7 interaction, we used AlphaFold 3 to predict the complex structure of AL7 and UBR7. The results indicated that the N-terminal PHD-associated ALFIN-Like (PAL) domain of AL7 is located at the interaction interface with UBR7 (Fig. 2, G and H). Further, glutathione *S*-transferase (GST) pull-down assays with purified GST-tagged PAL, variable motif (VM), and plant homeodomain (PHD) domains confirmed that UBR7 primarily interacts with the PAL domain of AL7 (Fig. 2I). Collectively, these findings demonstrate that UBR7 interacts with AL7 both in vivo and in vitro.

Next, we analyzed the proxitome of UBR7 using PL and identified 2119 proteins in the UBR7-TurboID samples. Among these, 577 proteins were recognized as enriched interactors based on a threshold of *P* < 0.05 and fold change (UBR7/citrine) ≥ 1.35 (fig. S5, A and B, and data S7). The previously confirmed UBR7 interactor H3 (*21*) was also identified in our MS dataset. AL7 was detected within the UBR7 proxitome as well (fig. S5B), supporting the reliability of the UBR7 PL data. Predictions for subcellular localization indicated that over half of the enriched UBR7 interactors are localized to the nucleus (fig. S5D and data S8). KEGG analysis further revealed that many of these proteins are involved in the ubiquitin system (fig. S5E and data S9). These findings reinforce the association between UBR7 and AL7 in the nucleus, implying UBR7’s role in the ubiquitination-mediated regulation of AL7.

### UBR7 mediates the ubiquitination and proteasomal degradation of AL7

**Fig. 3.**
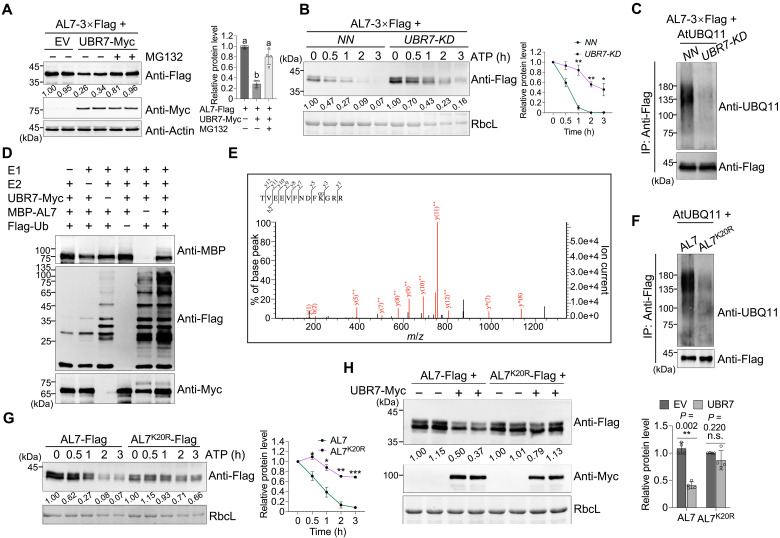
UBR7 mediates the degradation of AL7 via the 26*S* proteasome. (**A**) UBR7 decreases AL7 accumulation in a 26*S* proteasome–dependent manner. AL7-3×Flag was coexpressed with either the EV or UBR7-Myc through agroinfiltration of *N. benthamiana* leaves. At 36 hpi, 100 μM MG132 (+MG132) or an equal concentration of DMSO (−MG132) was further infiltrated into the preinfiltrated leaf regions. Leaf tissues were collected 12 hours after the treatment with DMSO or MG132 and subjected to immunoblot analysis. Actin served as the loading control. (**B**) A cell-free degradation assay was performed to test the effect of UBR7 on AL7 stability. AL7-3×Flag was agroinfiltrated into *NN* and *UBR7-KD* transgenic plants. At 2 days postinoculation (dpi), leaf samples were collected. The leaf extracts were treated with 10 mM ATP and 0.5 mM CHX for different time periods, as indicated above the panel. (**C**) AL7 ubiquitination levels decrease in *UBR7-KD* plants. (**D**) UBR7 ubiquitinates AL7 in vitro. (**E**) MS spectra of the identified peptides containing K20. Di-glycine (GG) above K20 indicates its potential as the ubiquitination site of AL7. (**F**) Mutation of the K20 site decreases the ubiquitination level of AL7. (**G**) A cell-free degradation assay analyzed the stability of AL7 and AL7^K20R^. (**H**) K20 ubiquitination contributes to UBR7-mediated AL7 degradation. For [(B) and (G)], a quantitative analysis of the degradation rates of AL7 or its derivatives is presented on the right. For (H), a quantitative analysis of the protein levels of AL7 and its AL7^K20R^ variant is illustrated on the right. Error bars represent means ± SD (*n* = 3 biological replicates). Asterisks above the corresponding line or bar chart indicate significant differences as determined by an unpaired two-tailed *t* test (n.s., not significant, *P* > 0.05; *P < 0.05; **P < 0.01; ***P < 0.001).

To investigate whether UBR7 mediates the proteasomal degradation of AL7, we coexpressed AL7 with UBR7 in *N. benthamiana* leaves. Immunoblot analysis revealed that AL7 accumulation was significantly reduced in the presence of UBR7, and this reduction was reversed by MG132 treatment (Fig. 3A). We also assessed the transcription levels of *AL7* in transient UBR7-overexpressing leaf tissues. The results indicated that UBR7 overexpression had no significant effect on the mRNA levels of *AL7* (fig. S6A), suggesting that UBR7 primarily regulates AL7 expression at the protein level in vivo.

To further characterize the role of UBR7 in regulating AL7 stability, we generated *UBR7* knockout (*UBR7-KO*) and knockdown (*UBR7-KD*) lines in *NN* plants (fig. S7, A and B). Both *UBR7-KO* and *UBR7-KD* affected normal plant growth, with *UBR7-KO* plants exhibiting more severe phenotypes (fig. S7C). This observation aligns with the previously reported dwarf phenotype in rice *UBR7-KO* mutants (*22*). Using TMV-GFP to inoculate various *N. benthamiana* lines, we confirmed UBR7’s negative regulatory role in *N*-mediated resistance against TMV (fig. S7, D and E).

A cell-free degradation assay revealed that AL7 degradation was significantly slower in extracts from *UBR7-KD* plants compared to control *NN* plants (Fig. 3B). In addition, *UBR7* knockdown did not lead to any significant change in *AL7* mRNA levels, indicating that UBR7 does not transcriptionally regulate AL7 expression (fig. S6B). However, immunoblot analysis showed that *UBR7* knockdown significantly reduced AL7 ubiquitination levels in vivo (Fig. 3C). To determine whether UBR7 functions as an E3 ubiquitin ligase mediating AL7 ubiquitination, we conducted an in vitro ubiquitination assay. The results demonstrated that, in the presence of ubiquitin, E1, and E2, UBR7 exhibited autoubiquitination activity, confirming its role as an E3 ligase. Moreover, UBR7 facilitated AL7 ubiquitination in the presence of E1 and E2, indicating that AL7 is a substrate of UBR7 (Fig. 3D). Collectively, these findings demonstrate that UBR7 directly targets AL7 for proteasomal degradation.

To identify the ubiquitination sites within AL7, we used two online tools, GPS-uber (https://gpsuber.biocuckoo.cn/online.php) and RUBI 1.0 (http://old.protein.bio.unipd.it/rubi/), to predict potential ubiquitination sites. Both servers identified K20 as a putative ubiquitination site (fig. S8A). We further enriched AL7-3×Flag from *N. benthamiana* using immunoprecipitation (IP), followed by LC-MS/MS analysis. The results indicated that AL7 undergoes ubiquitination at K20, K28, K121, and K142 (Fig. 3E and fig. S8B). All four residues are located within the PAL domain of AL7, which mediates its interaction with UBR7 (Fig. 2E). To validate the significance of K20, we mutated it to arginine, creating the AL7^K20R^ mutant. An in vivo ubiquitination assay demonstrated that the ubiquitination level of AL7^K20R^ was significantly reduced compared to wild-type AL7 (Fig. 3F). In addition, a cell-free degradation assay revealed that AL7^K20R^ was considerably more stable than wild-type AL7 (Fig. 3G). While UBR7 decreased the accumulation of wild-type AL7 and its derivative AL7^3KR^ (where K28, K121, and K142 were mutated to arginine), it had no significant effect on AL7^K20R^ accumulation (Fig. 3H and fig. S8C). These results underscore the critical role of K20 in UBR7-mediated ubiquitination and degradation of AL7.

### UBR7 genetically linked to AL7 during the *N*-mediated immune response

**Fig. 4.**
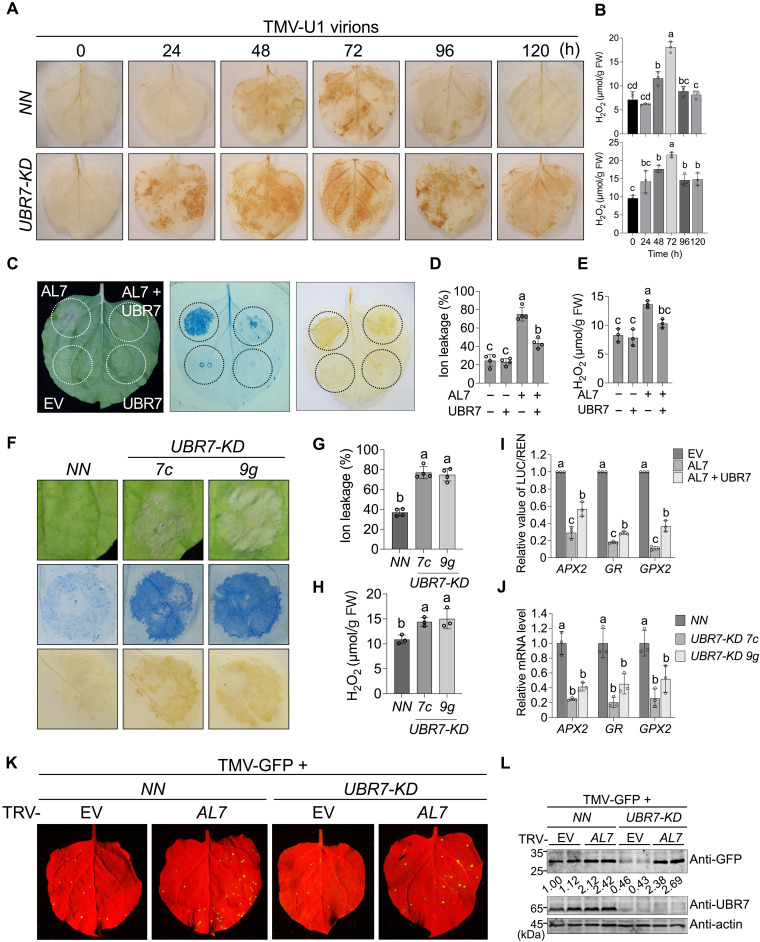
UBR7 negatively regulates AL7-induced ROS signaling during *N*-mediated resistance against TMV. (**A** and **B**) UBR7 negatively regulates ROS accumulation during the *N-*mediated immune response. TMV-U1 virions (200 ng) were rub-inoculated onto *NN* and *UBR7-KD* leaves. Inoculated leaves were collected for DAB staining (A) and H_2_O_2_ measurement (B) at different time points postinoculation. FW, fresh weight. (**C**) Overexpression of UBR7 impairs AL7-induced ROS production. (**D** and **E**) Ion leakage (D) and H_2_O_2_ levels (E) were measured in leaves as shown in (C). (**F**) Phenotypic observation of *NN* leaves transiently expressing AL7. *Agrobacterium* carrying AL7-Flag at optical density at 600 nm (OD_600_) = 0.1 was coinfiltrated with P19 into the leaves of *NN* and *UBR7-KD* plants. For (C) and (F), trypan blue and DAB staining were performed at 48 dpi. (**G** and **H**) Ion leakage (G) and H_2_O_2_ levels (H) were measured in leaves transiently expressing AL7 as displayed in (F). (**I**) Overexpression of UBR7 attenuates AL7’s transcriptional repressor activity. LUC activity was measured by normalizing it to the REN signal. (**J**) Knockdown of *UBR7* inhibits the expression of ROS scavenging genes. For [(D), (E), and (G) to (J)], error bars represent means ± SD [*n* = 4 biologically independent repeats for (D) and (G) and *n* = 3 for (E) and (H) to (J)]. Different letters in the chart indicate statistically significant differences among groups according to one-way analysis of variance (ANOVA) with Dunnett’s multiple comparison test (*P* < 0.05). (**K** and **L**) UBR7 negatively regulates *N*-mediated resistance against TMV in an AL7-dependent manner. TRV-EV– or TRV-*AL7–*infected *NN* or *UBR7-KD* plants were mechanically inoculated with GFP-tagged TMV-U1 virions, and photographs were taken under ultraviolet light at 3.5 dpi (K). TMV-U1-GFP accumulation was analyzed by immunoblotting with anti-GFP antibodies (L). All experiments were independently repeated at least twice with similar results. h, hours.

Our previous studies demonstrated that AL7 positively regulates *N*-mediated immune responses by promoting ROS accumulation (*17*). Since UBR7 mediates the ubiquitination and degradation of AL7 (Fig. 3), we analyzed whether UBR7 affects ROS accumulation during immune activation. 3,3′-diaminobenzidine (DAB) staining revealed that, in *NN* plants, ROS levels gradually increased following TMV infection, peaked at 72 hours postinoculation, and then declined (Fig. 4A). In contrast, ROS accumulation in *UBR7-KD* plants occurred more rapidly. It persisted longer than *NN* plants (Fig. 4, A and B). These findings revealed a “ROS wave” during *N*-mediated resistance against TMV, likely regulated by UBR7 through its effect on AL7’s role in promoting ROS production.

To clarify the genetic relationship between UBR7 and AL7, we transiently coexpressed AL7 with UBR7 in the leaves of *N. benthamiana*. Phenotypic observations indicated that UBR7 expression inhibited AL7-induced cell death and reduced ROS accumulation caused by AL7 overexpression (Fig. 4, C to E). In addition, the AL7^K20R^ mutant triggered stronger cell death and higher ROS levels compared to wild-type AL7, and UBR7’s effect on the AL7^K20R^ mutant was significantly diminished compared to its effect on wild-type AL7 (fig. S9, A and B). Furthermore, transient expression of AL7 in the leaves of *NN* and *UBR7-KD* plants demonstrated that *UBR7* knockdown exacerbated AL7-induced cell death and increased ROS accumulation (Fig. 4, F to H). These results indicate that UBR7 negatively regulates AL7-induced cell death and ROS accumulation.

We further analyzed the impact of UBR7 on AL7-regulated target genes, including *APX2*, *GR*, and *GPX2* (*17*). Through a dual-luciferase reporter assay, we found that AL7 expression significantly suppressed the transcription of these genes. Simultaneously, coexpression of UBR7 with AL7 reduced AL7’s ability to repress their expression (Fig. 4I). Moreover, the transcriptional repression activity of the AL7^K20R^ mutant was stronger than that of wild-type AL7 (fig. S9C). Quantitative reverse transcription polymerase chain reaction (qRT-PCR) analysis of *APX2*, *GR*, and *GPX2* expression in *NN* and *UBR7-KD* plants indicated that these genes were significantly down-regulated in *UBR7-KD* plants compared to the control *NN* plants (Fig. 4J). In summary, these findings suggest that UBR7 mediates the ubiquitination and degradation of AL7, thereby alleviating AL7-mediated repression of ROS-scavenging gene expression.

To further investigate UBR7’s role in regulating AL7 function during *N*-mediated immunity, we performed tobacco rattle virus (TRV)-based virus-induced gene silencing (VIGS) to down-regulate *AL7* in *NN* and *UBR7-KD* plants. TMV-GFP was inoculated into TRV-EV– or TRV-*AL7*–infected *NN* and *UBR7-KD* plants. At 3 days postinoculation (dpi), the results showed that, compared to nonsilenced *NN* plants, GFP fluorescence significantly increased in *AL7*-silenced leaves of *NN* plants, while it was notably reduced in *UBR7-KD* plants (Fig. 4, K and L), consistent with our previous findings (*17*, *20*). Furthermore, silencing *AL7* in *UBR7-KD* plants significantly compromised *N*-mediated resistance against TMV, as indicated by increased fluorescent spots reflecting TMV accumulation (Fig. 4, K and L). In addition, further silencing of AL7 in *UBR7-KD* plants using the TRV-VIGS vector partially reversed the down-regulation of ROS scavenging gene expression caused by *UBR7* knockdown, making it comparable to that in *NN* plants (fig. S10A). DAB staining revealed that *AL7* silencing also mitigates ROS accumulation in *UBR7-KD* plants in response to TMV infection, evidenced by the noticeably lighter brown coloration in the leaves of TRV-AL7–infected *UBR7-KD* plants compared to TRV-EV–infected *UBR7-KD* plants (fig. S10B). The down-regulation of *AL7* via the TRV-VIGS system was confirmed by qRT-PCR (fig. S11A). These results suggest that UBR7 operates upstream of AL7 in regulating *N*-mediated immunity.

### Phosphorylation promotes the ubiquitination and degradation of AL7

Our previous study revealed that AL7 undergoes phosphorylation during *N*-mediated immunity (*17*). To explore the potential relationship between AL7 phosphorylation and ubiquitination, we analyzed the temporal dynamics of these posttranslational modifications during *N*-mediated resistance against TMV. Our results showed that phosphorylation at the AL7 S174 site increased following TMV infection, peaking at 72 hours postinoculation (fig. S12A). Meanwhile, AL7 ubiquitination was markedly elevated at 96 hours postinoculation (fig. S12B), suggesting a functional interplay between AL7 phosphorylation and ubiquitination during *N*-mediated immunity.

**Fig. 5.**
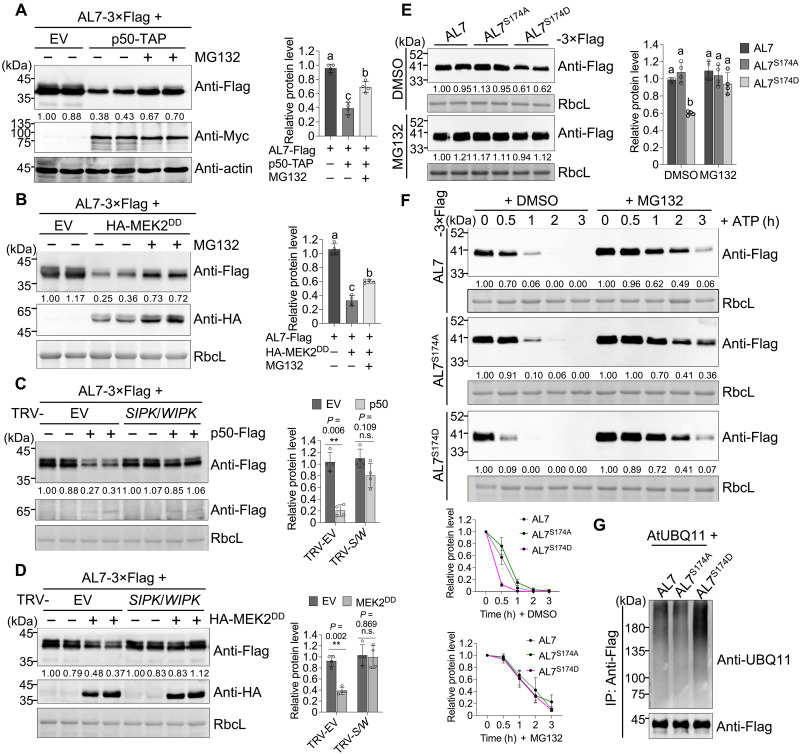
Activation of *N*-mediated immune responses promotes AL7 degradation in a phosphorylation-dependent manner. (**A** and **B**) AL7 was degraded in a 26*S* proteasome–dependent manner upon immune activation. AL7 was coexpressed with EV, tandem affinity purification (TAP) tag-fused p50 (p50-TAP) (A), and HA-MEK2^DD^ (B) via agroinfiltration in *NN* plants. At 32 hpi, 100 μM MG132 (+MG132) or an equal concentration of DMSO (−MG132) was further infiltrated into preinfiltrated leaf regions. Leaf tissues were collected 10 hours after the treatment with DMSO or MG132, followed by immunoblot analysis. (**C** and **D**) SIPK and WIPK were essential for AL7 degradation during immune responses. (**E**) Phosphorylation at S174 reduces AL7 protein accumulation in a 26*S* proteasome–dependent manner. AL7, AL7^S174A^, and AL7^S174D^ were transiently expressed in different regions of the same *N. benthamiana* leaves. At 36 hpi, 100 μM MG132 (+MG132) or an equal concentration of DMSO (−MG132) was further infiltrated into preinfiltrated leaf regions. Leaf tissues were collected 12 hours after treatment with DMSO or MG132, followed by immunoblot analysis. (**F**) A cell-free degradation assay was conducted to test the effect of S174 phosphorylation on AL7 stability. (**G**) S174 phosphorylation enhances AL7 ubiquitination. Quantitative analysis of protein levels of AL7 or its derivatives is presented to the right or below the corresponding immunoblot panels [(A) to (F)]. Error bars represent the means ± SD [*n* = 4 biological repeats for (A) to (E) and *n* = 3 for (F)]. Asterisks above the respective bar chart indicate significant differences determined by an unpaired two-tailed *t* test (n.s., *P* > 0.05; **P < 0.01). Different letters in the chart represent statistically significant differences among groups according to one-way ANOVA with Dunnett’s multiple comparison test (*P* < 0.05). Actin or RbcL served as the loading control.

Next, we analyzed changes in AL7 protein levels following immune activation. The results showed that immune activation through either p50 or constitutively activated mitogen-activated protein kinase kinase 2 (MEK2^DD^) reduced AL7 protein levels, which were largely restored by treatment with the proteasome inhibitor MG132 (Fig. 5, A and B). Since mitogen-activated protein kinases (MAPKs), salicylic acid–induced protein kinase (SIPK), and wounding-induced protein kinase (WIPK) mediate AL7 phosphorylation (*17*), silencing both *SIPK* and *WIPK* simultaneously mitigated the degradation of AL7 caused by immune activation, resembling the effect of MG132 treatment (Fig. 5, C and D). The down-regulation of *SIPK* and *WIPK* through the TRV-VIGS system was confirmed by qRT-PCR (fig. S11B). Furthermore, immunoblot analysis demonstrated that the protein accumulation of the phosphomimic mutant AL7^S174D^ was significantly lower compared to wild-type AL7 and the phospho-null mutant AL7^S174A^ (Fig. 5E, top). Notably, in the presence of MG132, there were no significant differences in the accumulation of AL7 and its derivatives (Fig. 5E, bottom). A cell-free degradation assay suggested that the degradation rate of AL7^S174D^ was faster than that of both AL7 and AL7^S174A^ (Fig. 5F). In addition, analysis of AL7 ubiquitination levels revealed that the in vivo ubiquitination level of AL7^S174D^ was remarkably higher than those of both AL7 and AL7^S174A^ (Fig. 5G). These results indicate that S174 phosphorylation promotes 26*S* proteasome–mediated degradation of AL7.

### UBR7 promotes reduced stability of phosphorylated AL7

To clarify whether UBR7 is necessary for the degradation of phosphorylated AL7, we first overexpressed UBR7 in leaves, both with and without silencing *SIPK* and *WIPK*. The results indicated that UBR7 overexpression enhanced AL7 degradation in nonsilenced leaves (fig. S13), consistent with the findings shown in Fig. 3A. However, UBR7’s ability to degrade AL7 was significantly compromised when both *SIPK* and *WIPK* were silenced (fig. S13), highlighting the relationship between AL7 phosphorylation and UBR7-mediated degradation.

**Fig. 6.**
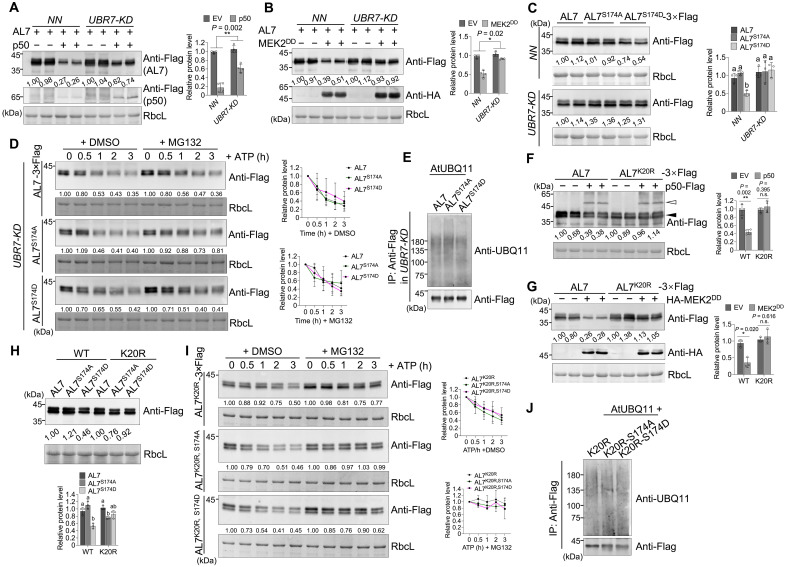
UBR7 is required for phosphorylation-driven degradation of AL7. (**A** and **B**) UBR7 is necessary for the degradation of AL7 during immune activation. Leaf tissues were collected at 42 hpi and analyzed by immunoblot. (**C**) The decrease in AL7 protein levels induced by S174 phosphorylation depends on UBR7. (**D**) A cell-free degradation assay was performed to evaluate the stability of AL7 or its variants in *UBR7-KD* plants. For (C) and (D), leaf samples were collected at 48 hpi. (**E**) The ubiquitination levels of AL7 or its derivatives in the leaves of *UBR7-KD* plants were analyzed. (**F** and **G**) K20 is essential for AL7 degradation during immune responses. Hollow and solid arrowheads indicate p50-Flag and AL7/AL7^K20R^-3×Flag, respectively. (**H**) The K20 mutation disrupts the S174 phosphorylation–induced instability of AL7. (**I**) A cell-free degradation assay was conducted to assess the stability of different AL7 variants. (**J**) The K20 mutation reduces the increase in AL7 ubiquitination levels induced by S174 phosphorylation. Quantitative analysis of protein levels of AL7 or its derivatives is shown to the right or below the corresponding immunoblot panels [(A) to (D) and (F) to (I)]. Error bars represent the means ± SD [*n* = 4 biological repeats for (A) to (C) and (F) to (H) and *n* = 3 for (D) and (I)]. Asterisks above the corresponding bar chart indicate significant differences determined by an unpaired two-tailed *t* test (n.s., *P* > 0.05; *P < 0.05; **P < 0.01). Different letters in the chart indicate statistically significant differences among different groups according to one-way ANOVA with Dunnett’s multiple comparison test (*P* < 0.05). Antibodies used are indicated to the right of corresponding immunoblot panels. RbcL served as the loading control. All experiments were independently repeated at least twice with similar results. h, hours.

Next, we analyzed the accumulation of AL7 protein in both *NN* and *UBR7-KD* plants during immune activation. The results showed that *UBR7* knockdown reduced the degradation of AL7 induced by immune activation (Fig. 6, A and B). Assessing the stability of AL7 and its derivatives in various *N. benthamiana* plants indicated that AL7^S174D^ had less accumulation compared to AL7 and AL7^S174A^ in *NN* plants. However, the protein levels of AL7^S174D^ were comparable to those of AL7 and AL7^S174A^ in *UBR7-KD* plants (Fig. 6C). A cell-free degradation assay demonstrated that the differences in degradation rates among AL7, AL7^S174A^, and AL7^S174D^ diminished in *UBR7-KD* plants (Fig. 6D). In addition, comparisons of ubiquitination levels revealed that AL7 and its derivatives exhibited similar ubiquitination levels in *UBR7-KD* plants (Fig. 6E).

To further characterize the interplay between AL7 phosphorylation and UBR7-mediated ubiquitination in regulating AL7 stability, we assessed whether immune activation influenced the accumulation of the AL7^K20R^ mutant, where the critical lysine residue (K) that UBR7 ubiquitinates was replaced with arginine (R) (Fig. 3). The results indicated that immune activation, triggered by either the p50 effector or MEK2^DD^, had no significant effect on AL7^K20R^ protein levels (Fig. 6, F and G). Further mutation of the S174 site in AL7^K20R^ to alanine (A) or aspartic acid (D) produced AL7^K20R,S174A^ and AL7^K20R,S174D^, respectively. Immunoblot analysis demonstrated that, unlike AL7 phosphorylation (AL7^S174D^), which decreased AL7 accumulation, the additional K20R mutation (AL7^K20R,S174D^) reversed the reduction in protein accumulation caused by the S174D mutation (Fig. 6H).

In addition, a cell-free degradation assay revealed no significant differences in the degradation rates of AL7^K20R^, AL7^K20R,S174A^, and AL7^K20R,S174D^ (Fig. 6I). Likewise, the ubiquitination levels of AL7^K20R^, AL7^K20R,S174A^, and AL7^K20R,S174D^ were mostly comparable (Fig. 6J), in contrast to the notable differences in ubiquitination levels observed for AL7, AL7^S174A^, and AL7^S174D^ in Fig. 5G. Overall, these results indicate that immune activation–induced AL7 phosphorylation promotes AL7 degradation in a UBR7-dependent manner.

### AL7 phosphorylation enhances UBR7-AL7 interaction

**Fig. 7.**
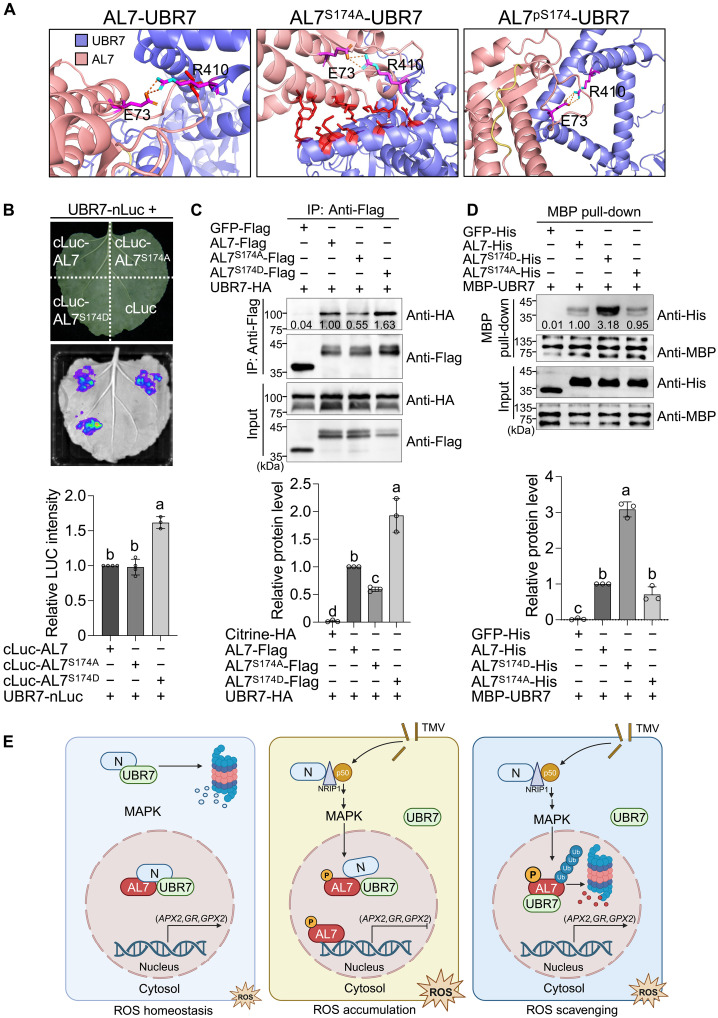
S174 phosphorylation enhances the interaction of AL7 with UBR7 and UBR7-AL7 signaling module. (**A**) Prediction of the interaction between UBR7 and AL7 or its derivatives using AlphaFold 3. (**B**) LCI assay to test the interaction between UBR7 and AL7 derivatives. UBR7-nLuc was coexpressed with cLuc-AL7, cLuc-AL7^S174A^, cLuc-AL7^S174D^, and cLuc alone in different regions of the same *N. benthamiana* leaves. Luminescence signals were recorded at 48 hpi. (**C**) Co-IP to analyze the interaction between UBR7 and AL7 derivatives. (**D**) MBP pull-down assay to detect the interaction between UBR7 and AL7 derivatives. MBP-UBR7 was incubated with AL7-His, AL7^S174A^-His, AL7^S174D^-His, and the negative control GFP-His. Input and pull-down products were subjected to immunoblot analysis with antibodies indicated on the right of the panels. (**E**) A proposed model for the phosphorylation-driven ubiquitination (Ub) switch that fine-tunes AL7-induced ROS signaling during *N*-mediated immune responses. NRIP1, N receptor-interacting protein 1.

To explore how AL7 phosphorylation facilitates its UBR7-mediated ubiquitination and degradation, we used the online AlphaFold 3 server to predict the complex structures of AL7, AL7^S174A^, and AL7^pS174^ (the phosphorylated form of AL7 at S174) with UBR7. The results indicated that the mutation at S174 alters the number of hydrogen bonds and steric hindrances between AL7 and UBR7. Specifically, AL7^S174A^ exhibited increased steric hindrance compared to AL7, while AL7^pS174^ showed an increase in the number of hydrogen bonds with UBR7 relative to AL7 (Fig. 7A), suggesting that AL7 phosphorylation may enhance its interaction with UBR7. We used LCI, Co-IP, and MBP pull-down assays to validate this hypothesis and analyze the interaction between UBR7 and AL7, AL7^S174A^, and AL7^S174D^. The results consistently demonstrated that AL7^S174D^ exhibited a stronger interaction with UBR7 than AL7 and AL7^S174A^ (Fig. 7, B to D). These findings indicate that AL7 phosphorylation enhances its interaction with UBR7, thereby promoting UBR7-mediated degradation of AL7.

## DISCUSSION

Aberrant activation of immune responses without pathogen infection or excessive immune responses can hinder plant growth and may even be lethal (*23*). ROS homeostasis is essential for balancing the trade-offs between plant growth and defense (*11*). On the basis of our previous findings (*17*, *20*) and this study, we propose a model for the UBR7-AL7 signaling module to precisely regulate ROS homeostasis during plant immunity. In the absence of pathogen infection, ROS-scavenging genes are expressed normally to maintain basal ROS levels (Fig. 7E, left). Upon perception of TMV infection, the N NLR activates the MAPK pathway, leading to the phosphorylation of AL7 at S174. Phosphorylated AL7 suppresses the expression of ROS-scavenging genes, including *APX2*, *GR*, and *GPX2*, thereby promoting ROS accumulation to initiate immune responses (Fig. 7E, middle) (*17*). Furthermore, S174 phosphorylation enhances the interaction between UBR7 and AL7, leading to UBR7-mediated ubiquitination and the subsequent degradation of AL7. This degradation restores the expression of ROS-scavenging genes, preventing excessive ROS accumulation (Fig. 7E, right). Our findings reveal how phosphorylation and ubiquitination collaborate to fine-tune ROS homeostasis at the transcriptional level during ETI responses.

UBR7 is a member of the UBR family protein, a unique class of E3 ubiquitin ligases characterized by their N-terminal UBR-box domain. In mammalian cells, UBR7 mediates monoubiquitination of the Histone 2B (H2B) protein to suppress tumorigenesis and metastasis (*24*, *25*). Moreover, UBR7 can promote the polyubiquitination of specific substrates, leading to their degradation (*26*–*28*). In plants, rice UBR7 has been reported to mediate H2B monoubiquitination, thereby regulating plant height (*22*). We previously identified *N. benthamiana* ubiquitin protein ligase E3 component N-recognin 7 (NbUBR7) and AL7 as N NLR immune receptor complex. However, it remains largely unclear whether UBR7 functions as an active E3 ubiquitin ligase and whether interactions occur among the various components of the N NLR immune receptor complex. In this study, we conducted in vivo and in vitro ubiquitination assays, demonstrating that NbUBR7 has E3 ubiquitin ligase activity (Fig. 3). TurboID-based PL unveiled multiple potential targets of UBR7, including AL7, which is studied here. This series of studies underscores the cross-talk among different components within the same N NLR immune receptor complex, constituting a sophisticated regulatory network for *N*-mediated immune signaling. We observed that the knockout of *UBR7* in *NN* leads to dwarfism similar to that reported in rice (*22*), which might be partially attributed to disrupted ROS homeostasis (Fig. 4, B and H).

The AL transcription factor family is unique to plants, with numerous studies demonstrating their crucial roles in regulating growth, development, and stress responses (*29*, *30*). Recently, *Zea mays* Alfin-like 14 (ZmAL14) was reported to be phosphorylated by sucrose non-fermenting-1-related protein kinase 2.2 (SnRK2.2), enhancing drought resistance in maize (*31*). In *Arabidopsis*, AL6 has been shown to undergo small ubiquitin-like modifier (SUMO) modification (SUMOylation), which increases its binding affinity to the target delay of germination-1 and reduces its ubiquitination levels, thereby protecting it from degradation via the 26*S* proteasome pathway (*32*). Conversely, the SUMOylation of AL3 appears to decrease its stability, as the SUMOylation site mutant AL3^K178R^ showed elevated protein accumulation (*33*). Furthermore, a systemic quantitative proteomic analysis of transcription factors identified extensive lysine ubiquitination in *Arabidopsis* AL family proteins, including AL1, AL2, AL3, and AL7 (*34*). In this study, we also demonstrated that AL7 in *N. benthamiana* undergoes ubiquitination, with K20 identified as a key ubiquitination site, suggesting a conserved mechanism of AL protein ubiquitination across different plant species. In addition, the interplay between SUMOylation and ubiquitination observed in AtAL6, which regulates seed dormancy and thermoinhibition (*32*), along with the cross-talk between phosphorylation and ubiquitination in NbAL7, which fine-tunes ROS homeostasis in plant immunity, highlights substantial interactions among the various posttranslational modifications of AL family proteins.

Previous studies have shown that regulating a transcription factor through phosphorylation and ubiquitination often enhances plant immunity. For example, the phosphorylation of OsWRKY31 has been reported to inhibit its ubiquitin-mediated degradation, thereby enhancing plant immunity (*35*). Shi *et al.* (*36*) found that phosphorylation and nonproteolytic ubiquitination of the ideal plant architecture 1 are required for its transcriptional activation and resistance against *Magnaporthe oryzae*. The turnover of the transcription factor MYC2, coupled with phosphorylation, is also critical for jasmonate-signaled plant immunity (*37*). While extensive studies have focused on regulating ROS homeostasis through the direct modulation of NADPH oxidases or the ROS scavenging system (*13*, *14*), the precise tuning of upstream transcription factors that govern these systems to manage ROS accumulation remains poorly understood. In this study, we demonstrate that phosphorylation drives the ubiquitination and subsequent degradation of AL7, thereby regulating the expression of downstream ROS-scavenging genes such as *APX2*, *GR*, and *GPX2*. Our findings reveal a distinct regulatory mechanism involving the interplay between phosphorylation and ubiquitination in modulating a specific transcription factor and ROS homeostasis during plant immune responses.

## MATERIALS AND METHODS

### Plasmid construction

To construct intron-spliced hairpin RNAi vectors for silencing *UBR7*, a 300–base pair segment from the target gene was selected using the online VIGS tool (https://vigs.solgenomics.net). The DNA fragment was inserted into the pSK-In vector at the Kpn I and Spe I sites. Subsequently, the DNA fragments that generate intron-hairpin RNAs were amplified and cloned into the pMDC32 plasmid at the Kpn I and Spe I sites using the Seamless Assembly Cloning Kit (Clone Smarter Technologies Inc., Houston, USA) according to the manufacturer’s instructions, resulting in the pMDC32-UBR7i vector. For CRISPR-based gene editing, a guide RNA for *UBR7* was designed using the online CRISPR RGEN Tools (www.rgenome.net/cas-designer/). The single guide RNA sequences were assembled into the BGK01 binary vector (Biogle, China) to create BGK01-UBR7.

To purify protein from *E. coli*, a cDNA fragment of UBR7 was cloned into a Bam HI– and Hind III–digested pMAL-C2X vector, enabling the expression of MBP-UBR7. Similarly, amplified cDNA fragments of UBR7, AL7, AL7^S174A^, or AL7^S174D^ were inserted into an Nde I– and Xho I–digested pET-30a vector to express His-tagged proteins in *E. coli*. Amplified full-length AL7 or its domains, PAL, VM, and PHD, were inserted into a Bam HI– and Hind III–digested pGEX-KG vector to express GST-tagged proteins in *E. coli*.

To express protein in *N. benthamiana*, entry vectors were constructed by inserting *UBR7* into the pDONOR207 vector (Invitrogen) using Gateway BP recombination. These vectors were then transferred to the corresponding destination vectors through Gateway LR recombination. The full-length cDNA of AL7 and its derivatives was cloned into the Kpn I– and Spe I–digested pMDC32 vector. The full-length cDNA of p50 was cloned into the Xho I– and Spe I–digested pTA7001 vector to generate dexamethasone (DEX)–inducible p50.

For BiFC assays, the full-length cDNA of *UBR7* was cloned into the pSPYCE vector (*38*) at the Bam HI site. *UBR7* was amplified and inserted into the Bam HI– and Sal I–digested pCAMBIA1300-nLuc vector (*39*) for split-Luc assays.

The primer sequences used for plasmid construction are detailed in table S1. All clones were confirmed through DNA sequencing.

### Generation of transgenic *N. benthamiana* plants

Transgenic *N. benthamiana* plants, comprising *UBR7-KD* and *UBR7-KO*, were generated through *Agrobacterium*-mediated transformation on a background of *N*-containing *N. benthamiana* plants (*NN*). *UBR7-KD* plants were obtained by transforming *NN* with *Agrobacterium* containing pMDC32-UBR7i, while *UBR7-KO* plants were produced by transforming *NN* with *Agrobacterium* containing BGK01-UBR7. Positive *UBR7-KD* plants were screened using qRT-PCR. Genomic DNA was extracted from *UBR7-KO* plants, followed by the amplification of the DNA fragment covering the guide RNA–targeted site. DNA sequencing was performed to analyze gene editing at the target locus of genomic *UBR7*.

### Plant growth conditions

*N. benthamiana* plants was grown in a greenhouse with a 15-hour light and 9-hour dark photoperiod at 23° to 24°C.

### BiFC assays

BiFC was conducted as previously described with minor modifications (*40*). *Agrobacterium* containing various BiFC vectors was mixed and infiltrated into *N. benthamiana* leaves. At 48 hpi, the infiltrated areas were collected for microscopy analysis using a Zeiss confocal microscope. Yellow fluorescent protein (YFP) fluorescence signals were excited at 488 nm.

### Firefly LCI assays

LCI assays were conducted as previously described (*39*). *Agrobacterium* containing various split-Luc vectors was mixed and infiltrated into *N. benthamiana* leaves. At 48 hpi, the infiltrated leaves were harvested, sprayed with luciferase substrate, and imaged using the NightSHADE LB985 In Vivo Plant Imaging System (Berthold, Germany).

### MBP pull-down assays

MBP pull-down assays were conducted as previously described (*41*). Briefly, 5 μg of MBP-UBR7 was mixed with 5 μg of GFP-His, AL7, AL7^S174A^, or AL7^S174D^ in a binding buffer containing 50 mM tris-HCl (pH 7.5), 100 mM NaCl, 5 mM dithiothreitol (DTT), 0.2% (v/v) glycerol, 0.6% (v/v) Triton X-100, and 1× proteinase inhibitor cocktail. This mixture was incubated with 20 μl of amylose resin (New England Biolabs, catalog number E8021V) for 3 hours at 4°C. The beads were collected by centrifugation at 800*g* for 1 min, followed by six washes with wash buffer containing 50 mM tris-HCl (pH 7.5), 600 mM NaCl, 0.2% (v/v) glycerol, and 0.6% (v/v) Triton X-100. The input and pull-down products were analyzed by immunoblotting with anti-MBP (EASYBIO, catalog number BE2020-100; 1:5000) and anti-His antibodies (EASYBIO, catalog number BE7001; 1:5000).

### Co-IP assays

Co-IP assays were performed as previously reported (*42*). Briefly, *Agrobacterium* mixtures expressing various protein combinations were infiltrated into *N. benthamiana* leaves. At 48 hpi, 3 g of infiltrated leaves were collected and ground into a powder using liquid nitrogen. Six milliliters of extraction buffer was added to the powder and mixed thoroughly. The mixture was incubated on ice for 45 min and then centrifuged at 3000*g* for 15 min at 4°C. The supernatant was incubated with 30 μl of anti-Flag beads for 3 hours at 4°C. Following incubation, the beads were collected by centrifugation at 800*g* for 1 min and washed six times with wash buffer [25 mM tris-HCl (pH 7.5), 1 mM EDTA, 50 mM NaCl, 10% (v/v) glycerol, and 0.1% (v/v) NP-40]. Input and IP products were analyzed by immunoblot using anti-Flag (Sigma-Aldrich, catalog number F1804; 1:5000), anti-HA (MBL, catalog number M180-3; 1:10000), and anti-Myc (MBL, catalog number M047-3; 1:10000) antibodies.

### Quantitative reverse transcription polymerase chain reaction

qRT-PCR was performed as previously described with minor modifications (*43*). Briefly, total RNA was extracted from *N. benthamiana* leaves using TRIzol (Invitrogen) according to the manufacturer’s instructions. Deoxyribonuclease I (Takara) was used to remove genomic DNA from 3 μg of RNA. Next, cDNA was synthesized through reverse transcription using Moloney murine leukemia virus (M-MLV) reverse transcriptase (Promega). A subsequent PCR test with 2× SsoFast EvaGreen Supermix (Bio-Rad) was conducted using cDNA as the template. The *Protein Phosphatase 2A* (*PP2A*) gene served as the internal control. Data analysis was performed using Bio-Rad CFX Manager (version 3.1). The primers used for qRT-PCR are listed in table S1.

### DAB staining

DAB staining was performed as previously described (*17*). Leaves were incubated in a staining buffer [DAB solution (1 mg/ml) made by dissolving 25 mg of DAB powder in 25 ml of deionized water] for 6 hours in the dark at room temperature. They were then destained by boiling in 95% ethanol for 10 min. The destained leaves were subsequently transferred to 75% ethanol for photography.

### Trypan blue staining

Trypan blue staining was conducted as previously reported (*44*). Leaves were immersed in the staining buffer (composed of 10 ml of deionized water, 10 ml of glycerol, 10 ml of lactic acid, 10 ml of water-saturated phenol, and 40 ml of absolute ethanol), boiled for 10 min, and incubated at room temperature for 5 hours. The leaves were then destained three times in chloral hydrate solution (2.5 g/ml).

### Viral inoculation

Viral inoculation was conducted according to previously described procedures (*45*), and hundred nanograms of TMV-U1 or TMV-U1-GFP virions were diluted in 0.01 M phosphate-buffered saline buffer and mechanically inoculated onto the leaves of 4- to 6-week-old *N. benthamiana* plants.

### In vitro ubiquitination assays

The ubiquitination assay was performed using a reconstituted *E. coli*–based system as previously described (*46*). The plasmids pCDFDuet-MBP-AL7-UBA1-S, pACYCDuet-UBR7-MYC-UBC8-S, and pET28a-Flag-Ub were cotransformed into *E. coli* BL21 (DE3). The bacteria were cultured in liquid LB medium. When the absorbance at optical density at 600 nm (OD_600_) reached 0.5, 0.5 mM isopropyl-β-d-thiogalactopyranoside was added to induce the expression of recombinant proteins. The cultures were then incubated at 28°C for 10 hours. Protein ubiquitination was assessed by immunoblotting using anti-MBP, anti-Flag, and anti-MYC antibodies.

### In vivo ubiquitination assays

In vivo ubiquitination assays were conducted as previously reported with minor modifications (*41*). An *Agrobacterium* mixture expressing AL7 or its derivatives, along with AtUBQ11, was infiltrated into *NN* or *UBR7-KD* leaves. The agroinfiltrated leaves were harvested 48 hpi and ground into a powder in liquid nitrogen. Total proteins were extracted using an extraction buffer composed of 25 mM tris-HCl (pH 7.5), 1 mM EDTA, 150 mM NaCl, 10% (v/v) glycerol, 10 mM DTT, 0.1% (v/v) NP-40, 2% (m/v) polyvinyl pyrrolidone 40, a 1× proteinase inhibitor cocktail, 10 mM *N*-ethylmaleimide (Sigma-Aldrich, catalog number E1271), 10 nM ubiquitin aldehyde (EMD Millipore Corporation, catalog number 662056), 1 mM phenylmethylsulfonyl fluoride (PMSF), and 50 μM MG132. The mixture was then enriched using anti-Flag beads (GNI, catalog number GNI4510-FG) at 4°C for 3 hours. The beads were subsequently washed three times with wash buffer I, which contained 25 mM tris-HCl (pH 7.5), 1 mM EDTA, 150 mM NaCl, 10% (v/v) glycerol, 0.1% (v/v) NP-40, a 1× proteinase inhibitor cocktail, 10 mM *N*-ethylmaleimide, 10 nM ubiquitin aldehyde, 1 mM PMSF, and 50 μM MG132. They were then washed another three times with wash buffer II, consisting of 25 mM tris-HCl (pH 7.5), 1 mM EDTA, 150 mM NaCl, 10% (v/v) glycerol, 0.1% (v/v) NP-40, 10 mM *N*-ethylmaleimide, 10 nM ubiquitin aldehyde, and 1 mM PMSF. The immunoprecipitated products were then analyzed by immunoblotting using an anti-AtUBQ11 antibody.

### Cell-free protein degradation assays

Cell-free protein degradation assays were conducted as previously described (*47*), with minor modifications. Total proteins were extracted from *N. benthamiana* leaves expressing AL7, AL7^S174A^, or AL7^S174D^ using a native buffer [50 mM tris-HCl (pH 8.0), 10 mM EDTA (pH 8.0), 0.5 M sucrose, 1 mM MgCl_2_, and 5 mM DTT]. The extracts were supplemented with 0.5 mM cycloheximide (CHX) and 10 mM ATP and then incubated at room temperature for various periods. Protein levels were assessed through immunoblot analysis using an anti-Flag antibody.

### Dual-luciferase reporter assays

The dual-luciferase reporter assay was performed using the previously described method (*17*). AL7 or AL7^K20R^ was coexpressed with a Luc cassette driven by the native promoter of *APX2*, *GR*, and *GPX2* in 4-week-old *N. benthamiana* leaves. At 3 dpi, the ratios of ratio of Firefly luciferase (LUC) to Renilla luciferase (REN) (LUC/REN) were measured using dual-luciferase assay reagents (Promega, Madison, USA) on a GloMax 20/20 luminometer (Promega) following the manufacturer’s instructions.

### Ion leakage measurement

Ion leakage measurement was conducted as previously described with minor modifications (*48*). Discs cut from infiltrated leaves were floated in deionized water for 1 hour at room temperature. The first measurement was taken using a DDS-12DW conductivity meter (BANTE) and recorded as S1. The discs were then boiled for 45 min, followed by 1 hour of flotation at room temperature. The second measurement was taken and recorded as S2. The ratio of S1 to S2 was calculated and presented as ion leakage.

### H_2_O_2_ measurement

Thirty milligrams of leaves were collected and ground into a powder using liquid nitrogen. Subsequently, 200 μl of 25 mM sodium phosphate buffer was used to suspend the sample. After centrifugation at 13,500*g* for 15 min at 4°C, the H_2_O_2_ level was assessed using the Amplex Red Hydrogen Peroxide/Peroxidase Assay Kit on a Tecan Spark 20M, adhering to the manufacturer’s instructions.

### Sample preparation for PL analysis

Leaf samples for PL analysis were prepared as previously described, with minor modifications (*49*, *50*). Briefly, 4- to 6-week-old *NN* leaves were infiltrated with *Agrobacterium* containing various constructs, as indicated in figs. S2E and S5A. At 40 hpi, 200 mM biotin was infiltrated into the preinfiltrated leaves, followed by an 8-hour incubation. When the p50 effector was used to activate *N*-mediated immunity (+p50), 200 mM biotin and 30 μM DEX were coinfiltrated into *NN* leaves. For the control group (−p50), the same volume of dimethyl sulfoxide (DMSO) solvent (without dexamethasone) was used for infiltration.

Infiltrated leaves were harvested and ground into powders using liquid nitrogen. Protein extraction from the leaf powder was performed with radioimmunoprecipitation assay buffer and a Zeba Spin Desalting Column (Thermo Fisher Scientific, catalog number 89893). After quantification using the Bradford assay, equal amounts of total proteins and streptavidin beads (Invitrogen, catalog number 65001) were incubated for a minimum of 16 hours at 4°C. The streptavidin beads were then washed sequentially with buffer I [2% (w/v) SDS], buffer II [50 mM Hepes, 500 mM NaCl, 1 mM EDTA, 0.1% (w/v) deoxycholic acid, and 1% Triton X-100], buffer III [10 mM tris-HCl, 250 mM LiCl, 1 mM EDTA, 0.1% (w/v) deoxycholic acid, and 1% (v/v) IGEPAL-630], and 50 mM tris-HCl (pH 7.5) twice, followed by six washes in 50 mM ammonium bicarbonate. The streptavidin-enriched products were flash-frozen in liquid nitrogen and stored at −80°C.

### Data-independent acquisition analysis

Biotinylated proteins enriched on streptavidin beads were enzymatically digested with trypsin (Thermo Fisher Scientific, catalog number 90058) overnight at 37°C. The resulting digested peptides were collected using 10,000-Da molecular weight cutoff Hydrosart ultrafiltration products from VI-VACON 500 (Sartorius Stedim Biotech, catalog number VN01H02). An equal amount of peptides were dissolved in 50 mM triethylammonium bicarbonate (Sigma-Aldrich, catalog number T7408) and analyzed using a Q-Exactive high-resolution mass spectrometer (Thermo Fisher Scientific, Waltham, MA, USA) to evaluate peptide quality.

The digested peptide mixtures were analyzed using LC-MS/MS on a Q-Exactive high-resolution mass spectrometer (Thermo Fisher Scientific, Waltham, MA, USA) equipped with a nanoACQUITY high-performance liquid chromatography (Waters, Milford, MA, USA). The peptides were injected onto a trap column (Acclaim PepMap, 75 μm by 2 cm, 3 μm, C18, 100 Å, Thermo Fisher Scientific, Waltham, MA, USA) and separated using an analytical column (Aqua, 100 μm by 15 cm, 3 μm, C18, 125 Å, Phenomenex, Los Angeles, CA, USA). The flow rate was set at 400 nl/min. The peptides were eluted with a 125-min gradient elution method, where mobile phase A consisted of 0.1% formic acid (FA) in water and mobile phase B contained 0.1% FA in acetonitrile. MS measurement scans were acquired in the range of 300 to 1800 mass/charge ratio (*m*/*z*) at a resolution of 70,000. For higher-energy collisional dissociation, the dynamic exclusion time was set to 20 s, and the whole scan resolution for the 10 most significant values in an ion trap was 17,500.

Mascot (version 2.4; Matrix Science, London, UK) and Scaffold software (version 3.6.5, Proteome Software, Portland, USA) were used to analyze the resulting data for protein identification and quantification. Mascot was used to identify proteins, including common contaminants, as well as proteins identified solely by peptides matching a database of reverse peptide sequence decoys, which were excluded from our data. Furthermore, proteins identified by fewer than two unique peptides and with a *P* < 0.05 from the Mann-Whitney *U* test with Benjamini-Hochberg correction were also removed from the lists. The remaining proteins were considered “value detected.”

### Interaction network construction

Proteins within the AL7-proximal protein-protein interaction (PPI) network primarily underwent filtration based on a cutoff of *P* < 0.05 from the Mann-Whitney *U* test with Benjamini-Hochberg correction, alongside fold changes (AL7-TurboID/NLS-citrine-TurboID) ≥1.5, which is considered high confidence. A subset of *Arabidopsis thaliana* orthologs corresponding to the input of *N. benthamiana* proteins was identified, and interactions among these proteins were predicted using the GeneMANIA online platform (https://genemania.org). The resulting PPI network was visualized and refined using Adobe Illustrator (version 26.3.1).

### MS data analysis

To analyze the protein levels of “value-detected” proteins, we obtained average values from three biological replicates. We normalized them against the distribution of biotinylated proteins about the NLS-citrine-TurboID or citrine-TurboID group. The log_2_ (normalized ratio) was calculated for subsequent analysis. Proximal proteins that met a fold change threshold of ≥1.5 or 1.35 were considered closely associated with the bait protein. The subcellular localization of significantly enriched proteins was predicted using the BUSCA web server (http://busca.biocomp.unibo.it). Volcano plots, KEGG bubble plots, and heatmaps were generated using RStudio software with the R programming language, illustrating grouped pathways for significantly enriched proteins.

### Protein structure and interaction prediction

AlphaFold 3 (https://golgi.sandbox.google.com/) was used to examine PPIs, followed by PyMOL (www.pymol.org/) for structural alignment, analysis, and annotation. The amino acid sequence of the target protein was retrieved from UniProt and entered into AlphaFold 3. The sequences of the interacting proteins were also processed similarly in AlphaFold 3 to simulate the unmodified protein structure and its interaction with binding partners.

To analyze proteins with posttranslational modifications, the modified protein sequence was reentered into AlphaFold 3. The specific type of modification was assigned to the corresponding amino acid residue, and the interacting protein sequences were incorporated to generate the modified protein structure and interaction model. The predicted interaction structures were downloaded from AlphaFold 3 and imported into PyMOL for further analysis. The pre- and postmodification structures were aligned at the N-terminal for direct comparison. Atomic-scale details of the modified sites and residue-level interaction surfaces were examined to assess the impact of phosphorylation on PPIs. Last, potential interaction forces were annotated in PyMOL to highlight structural and functional changes induced by phosphorylation.

### Quantification and statistical analysis

The statistical significance of the results for ion leakage and H_2_O_2_ measurements, dual-luciferase reporter, qRT-PCR, and immunoblot is determined by Student’s *t* test [not significant (n.s.), *P* > 0.05; *P < 0.05; **P < 0.01; ***P < 0.001] or Dunnett’s multiple comparison test (different letters indicate significant differences, *P* < 0.05). For immunoblot quantification analysis, band intensities from three or four independent replicates are measured using ImageJ software (National Institutes of Health, Bethesda, Maryland, USA).
