# Supplementary material for: A phosphorylation-driven ubiquitination switch fine-tunes Alfin-like 7–induced ROS signaling in plant immunity
Source: Sci Adv. 2025 Jun 13;11(24):eadw7554. doi: 10.1126/sciadv.adw7554 (PMC12164958; doi:10.1126/sciadv.adw7554)
Supplement: Supplementary file 1 — Figs. S1 to S13 Table S1 Legends for data S1 to S9 [file sciadv.adw7554_sm.pdf]

Science Advances
AAAS

Supplementary Materials for

A phosphorylation-driven ubiquitination switch fine-tunes Alfin-like 7–induced ROS signaling in plant immunity

Dingliang Zhang *et al.*

Corresponding author: Yongliang Zhang, cauzhangyl@cau.edu.cn;
Savithramma P. Dinesh-Kumar, spdineshkumar@ucdavis.edu

Sci. Adv. **11**, eadw7554 (2025)
DOI: 10.1126/sciadv.adw7554

The PDF file includes:

Figs. S1 to S13
Table S1
Legends for data S1 to S9

Other Supplementary Material for this manuscript includes the following:

Data S1 to S9

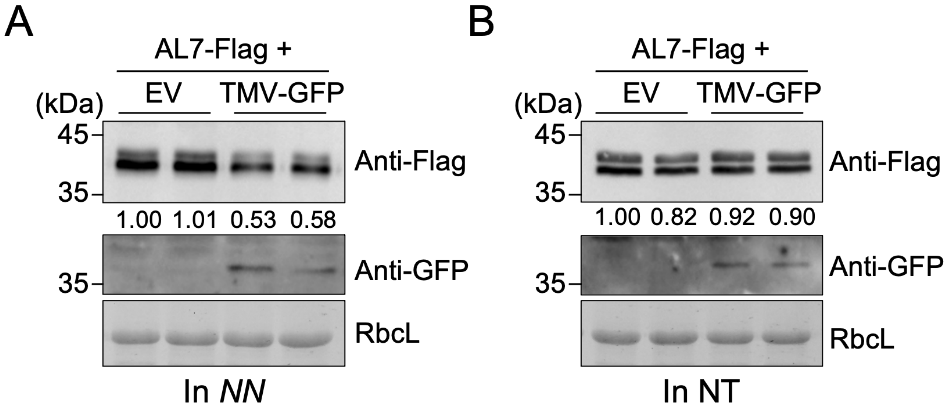

Fig. S1. Analysis of AL7 stability in *NN* (A) and NT (B) *N. benthamiana* plants during TMV infection. AL7 was co-expressed with either an empty vector (EV) or TMV-GFP in *NN* and NT *N. benthamiana* plants. Leaf samples were collected 48 hours post-infiltration for immunoblot analysis. RbcL served as the loading control.

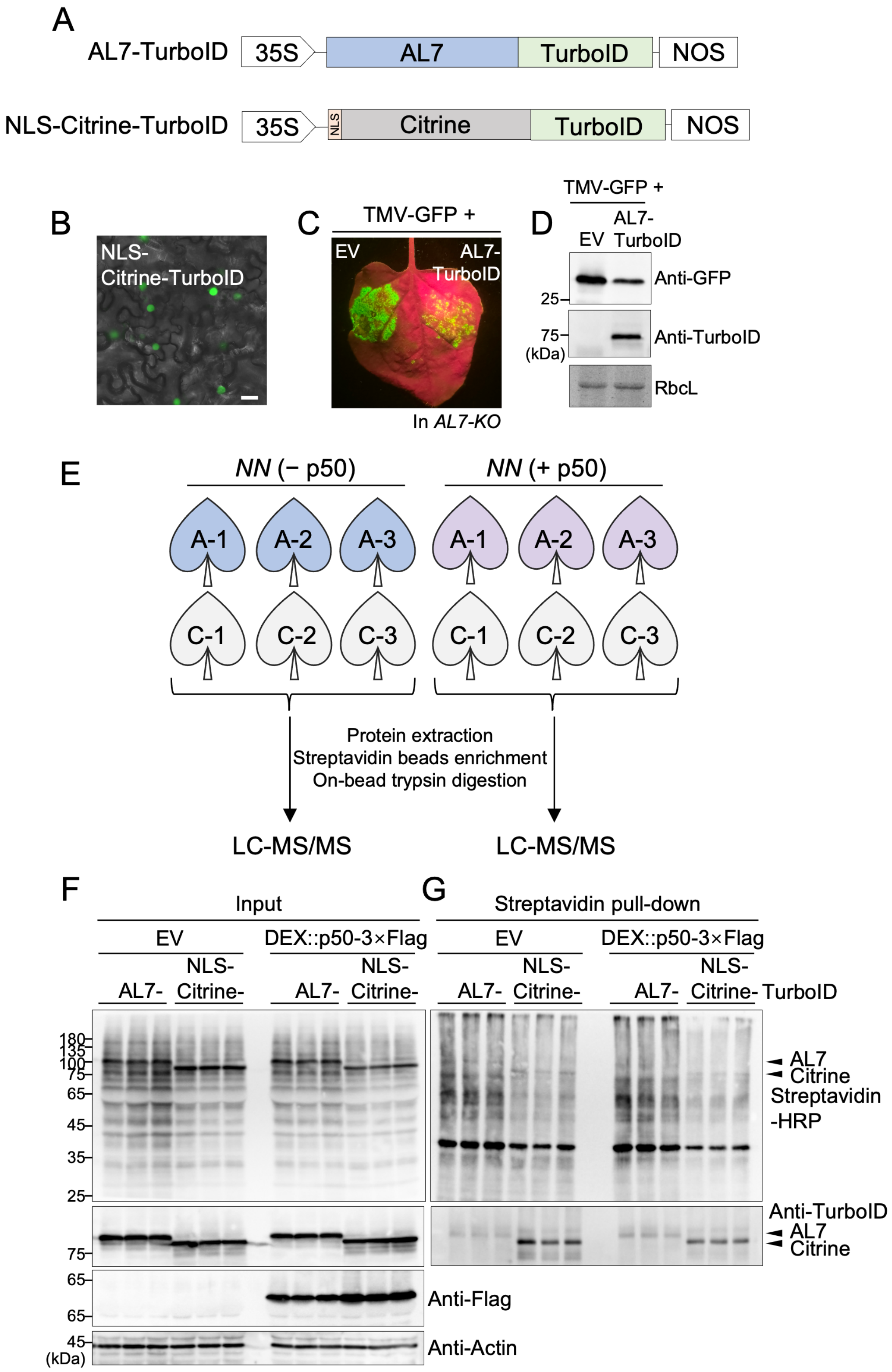

Fig. S2. Proxitome profiling of AL7 protein using TurboID-based proximity labeling. (**A**) Diagram of the constructs used for proxitome profiling of the AL7 protein. NLS refers to the nuclear localization signal. (**B**) Confocal microscopy analysis to validate the nuclear localization of NLS-Citrine-TurboID. (**C−D**) The fusion of

TurboID to the C-terminus of AL7 retains its ability to rescue *N*-mediated resistance against TMV in *AL7-KO N. benthamiana* plants. (**E**) Workflow for the PL analysis of AL7 protein in this study. *Agrobacterium* mixtures containing fusions, with (+ p50) or without p50 (− p50), were co-infiltrated into the leaves of *N. benthamiana NN* plants under the control of the DEX-inducible promoter. At 34 hpi, 200 μM biotin and 30 μM DEX were infiltrated into the pre-infiltrated leaves. After an 8-hour incubation, the infiltrated leaves were collected for subsequent processing as indicated on the panel. Each treatment was performed with three independent biological replicates (n = 3 plants for each replicate). A and C represent AL7-TurboID and NLS-Citrine-TurboID, respectively. (**F**) Immunoblot analysis is performed to validate protein expression and biotinylation in the infiltrated leaves, as shown in panel E. (**G**) Immunoblot analysis is conducted to verify the enrichment of biotinylated proteins. Streptavidin-HRP or antibodies used for immunoblot analysis are indicated on the right of the corresponding panels.

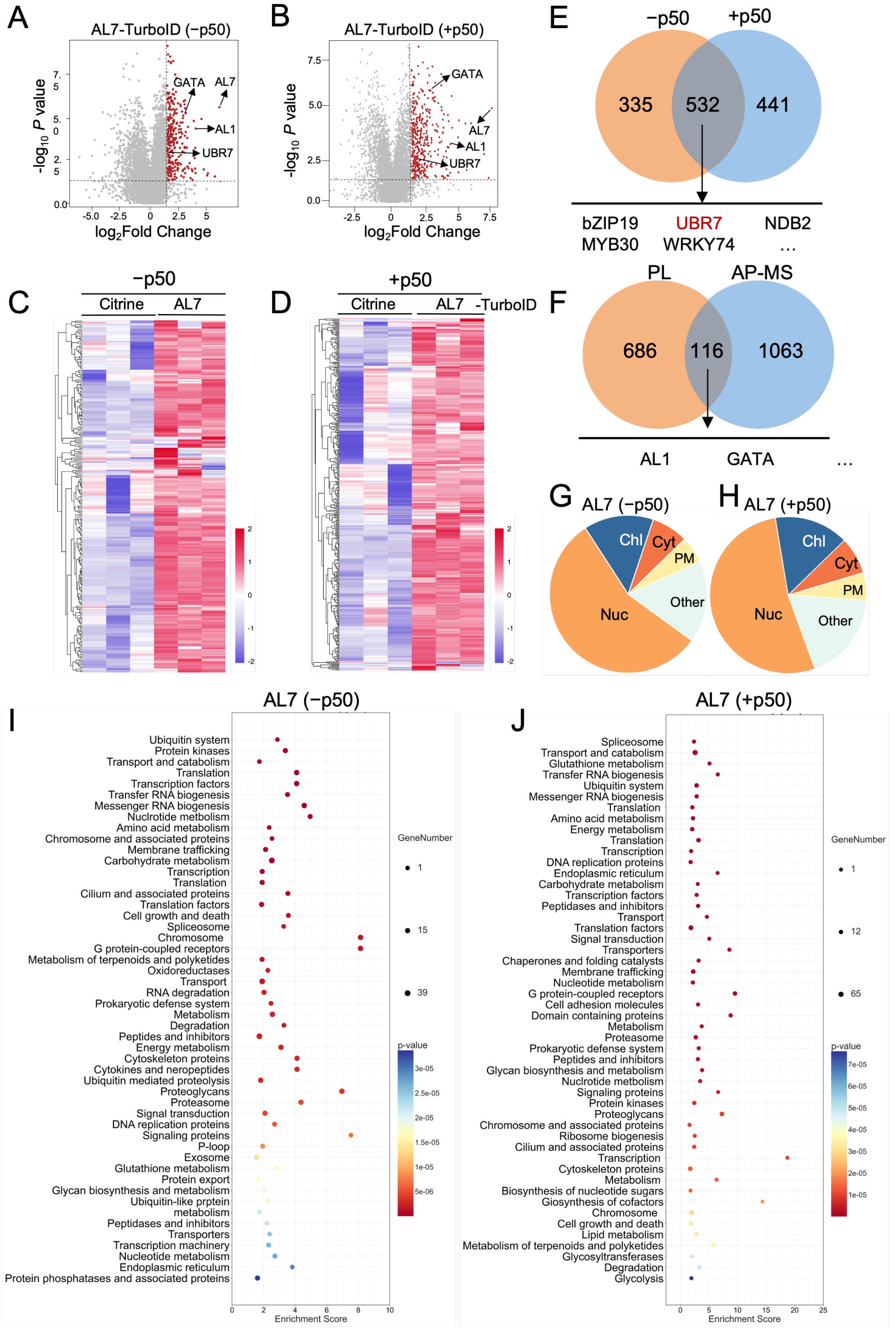

Fig. S3. Bioinformatic analysis of biotinylated proteins enriched in AL7-TurboID purification. (**A–B**) Volcano plot showing proteins identified by TurboID-based PL in three biologically independent experiments (n = 3 plants for each experiment). The mean $\log_2$ fold change of three replicates between the control group NLS-Citrine-

TurboID and AL7-TurboID in the presence of TMV p50 (+ p50) or absence of TMV p50 (− p50) was plotted against the $-\log_{10} P$ value. Values are provided in Data S1. (**C–D**) Heatmap based on significantly upregulated data from individual samples of NLS-Citrine-TurboID (n = 3) compared with AL7-TurboID (n = 3) in the presence or absence of TMV p50. Heatmaps and the protein clustering tree on the left were generated using R studio (AGPL v3). The relative protein expression values range from the highest (red) to the lowest (blue), as indicated by the lower middle of the color scale bar (-2.0 to +2.0). Values are provided in Data S2. (**E**) Venn diagram showing overlaps among AL7-TurboID in the presence of TMV p50 (+ p50) or absence of TMV p50 (− p50). The UBR7 protein represents the protein investigated further in this study. Values are provided in Data S3. (**F**) Venn diagram showing overlaps among the AL7 proxitome in our study and the AL7 AP-MS results by Su et al (*19*). Values are provided in Data S4. (**G–H**) Pie charts showing the proportion of proteins identified in PL experiments with different subcellular localizations. The significantly enriched proteins from PL experiments were analyzed using the online BUSCA web server. Nuc, nucleus; Cyt, cytoplasm; Chl, chloroplast; PM, plasma membrane. Values are provided in Data S5. (**I–J**) Kyoto Encyclopedia of Genes and Genomes (KEGG) enrichment analyses of the significantly enriched proteins identified in AL7-TurboID in the presence of TMV p50 (+ p50) or absence of TMV p50 (− p50) (Fold change ≥ 1.5, $P < 0.05$). The count value represents the number of proteins enriched in the relevant pathway. Values are provided in Data S6.

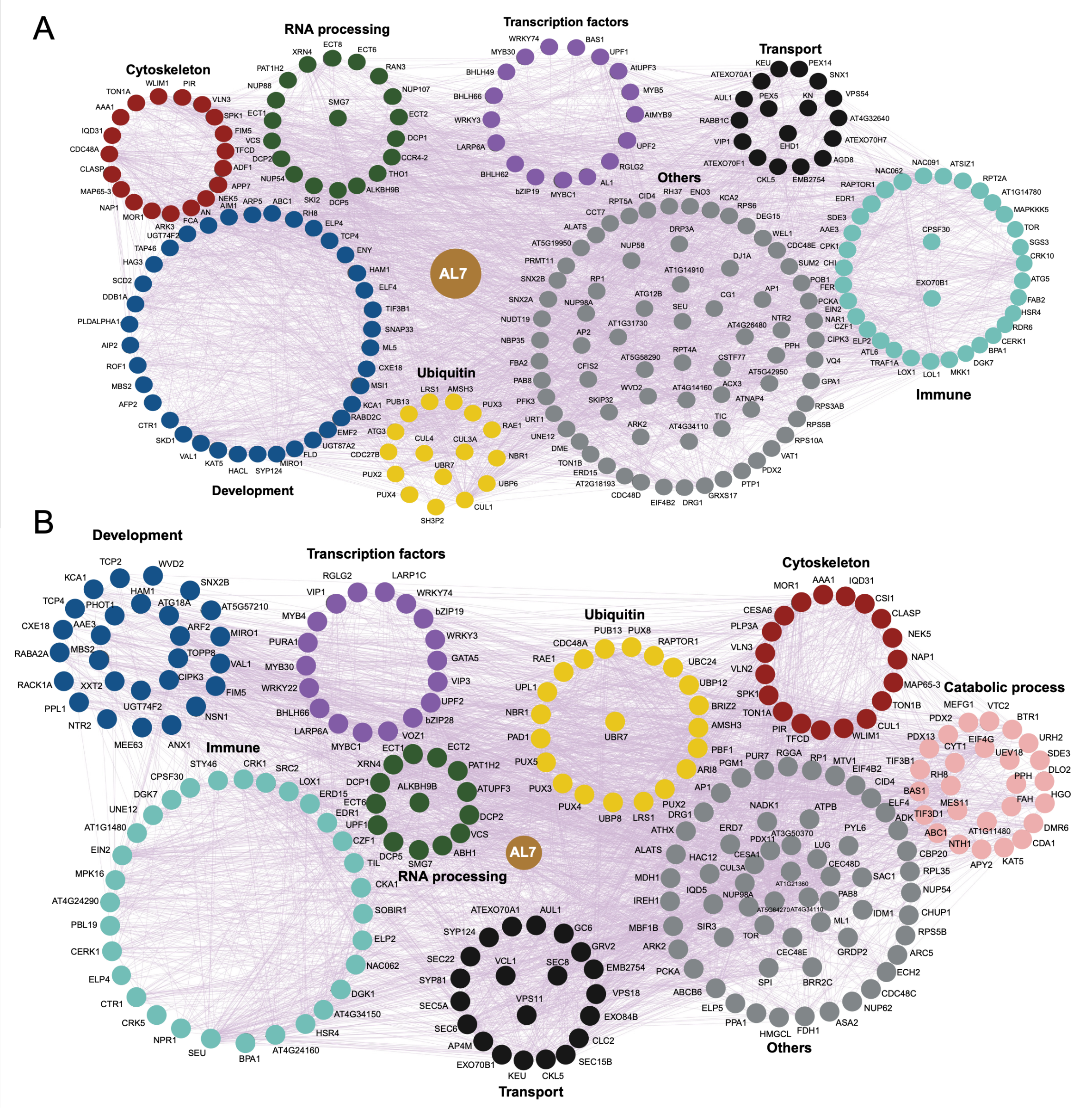

Fig. S4. Schematic representation of the AL7 proximal interactome. The AL7 protein interaction networks are shown in the absence of p50 (**A**) and in the presence of p50 (**B**), respectively. The clusters are color-coded as indicated. Nodes represent individual proteins, and their names are provided. Edges between the nodes represent interactions based on GeneMania. Clusters are labeled according to KEGG enrichment results obtained from the KEGG website (https://www.kegg.jp).

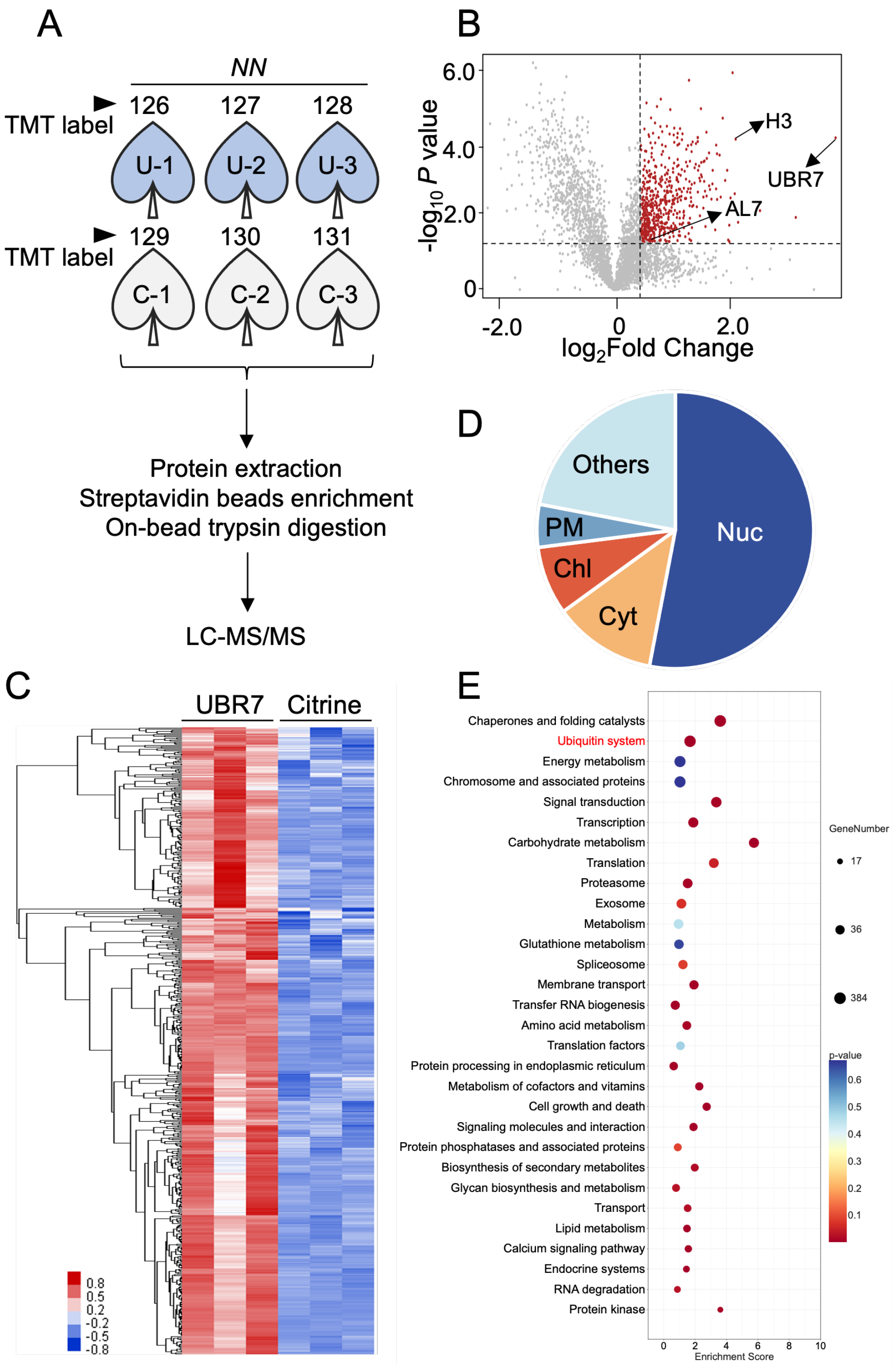

Fig. S5. TurboID-based PL analysis of UBR7 protein. (A) Workflow for the PL analysis of UBR7 protein in this study. *Agrobacterium* expressing UBR7-TurboID or Citrine-TurboID was infiltrated into the leaves of *N. benthamiana NN* plants. At 40 h post-infiltration (hpi), 200 μM biotin was introduced into the pre-infiltrated leaves. After an 8-hour incubation, the infiltrated leaves were collected for subsequent processing, as indicated in the panel. Each treatment was conducted with three

independent biological replicates (n = 3 plants for each replicate). U and C represent UBR7-TurboID and Citrine-TurboID, respectively. The numbers 126 to 131 above the leaf schematic indicate the 6-plex TMT reporter ion masses at m/z 126 to 131. (**B**) Volcano plots showcasing the identified proximal proteome of UBR7. Colored dots represent significantly enriched proximal proteins (Fold change ≥ 1.35). Data are derived from three biologically independent experiments (n = 3) with $P < 0.05$. Proteins identified in the control Citrine-TurboID samples were used to filter UBR7 candidates. Values are provided in Data S7. (**C**) Heatmap displaying the significantly enriched proteins proximal to UBR7. The protein clustering tree is indicated on the left. The scale shows a $\log_2$ Fold change of protein abundance on the left, and the color key indicates protein expression levels: blue for low, red for high, and white for medium. (**D**) Pie charts illustrating the proportion of proteins identified in PL experiments with different subcellular localizations. The significantly enriched proteins from UBR7-TurboID PL experiments were analyzed using the online BUSCA web server. Nuc indicates nucleus; Cyt indicates cytoplasm; Chl indicates chloroplast; PM stands for plasma membrane. Values are provided in Data S8. (**E**) Kyoto Encyclopedia of Genes and Genomes (KEGG) enrichment analyses of the significantly enriched proteins identified in the UBR7-TurboID (Fold change ≥ 1.35, $P < 0.05$). The count value represents the number of proteins enriched in the relevant pathway. Values are provided in Data S9.

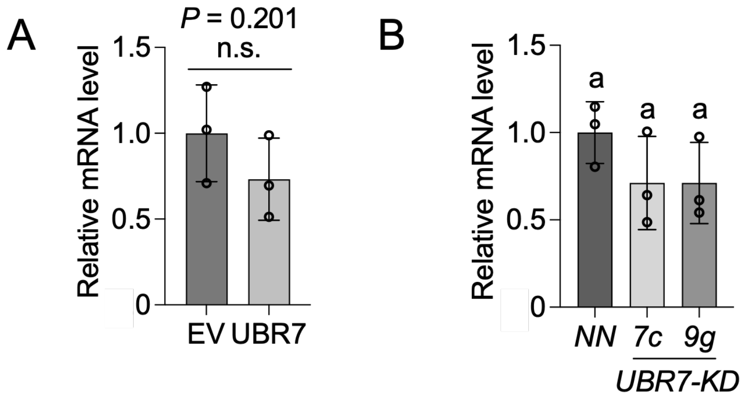

Fig. S6. UBR7 does not significantly affect the transcription of *AL7*. (**A**) qRT-PCR analysis of *AL7* mRNA levels in response to UBR7 overexpression. (**B**) qRT-PCR analysis of *AL7* mRNA levels in *UBR7-KD N. benthamiana* plants. Error bars indicate mean ± SD (n = 3 biological repeats; n.s. = not significant, $P > 0.05$, paired two-tailed *t*-test).

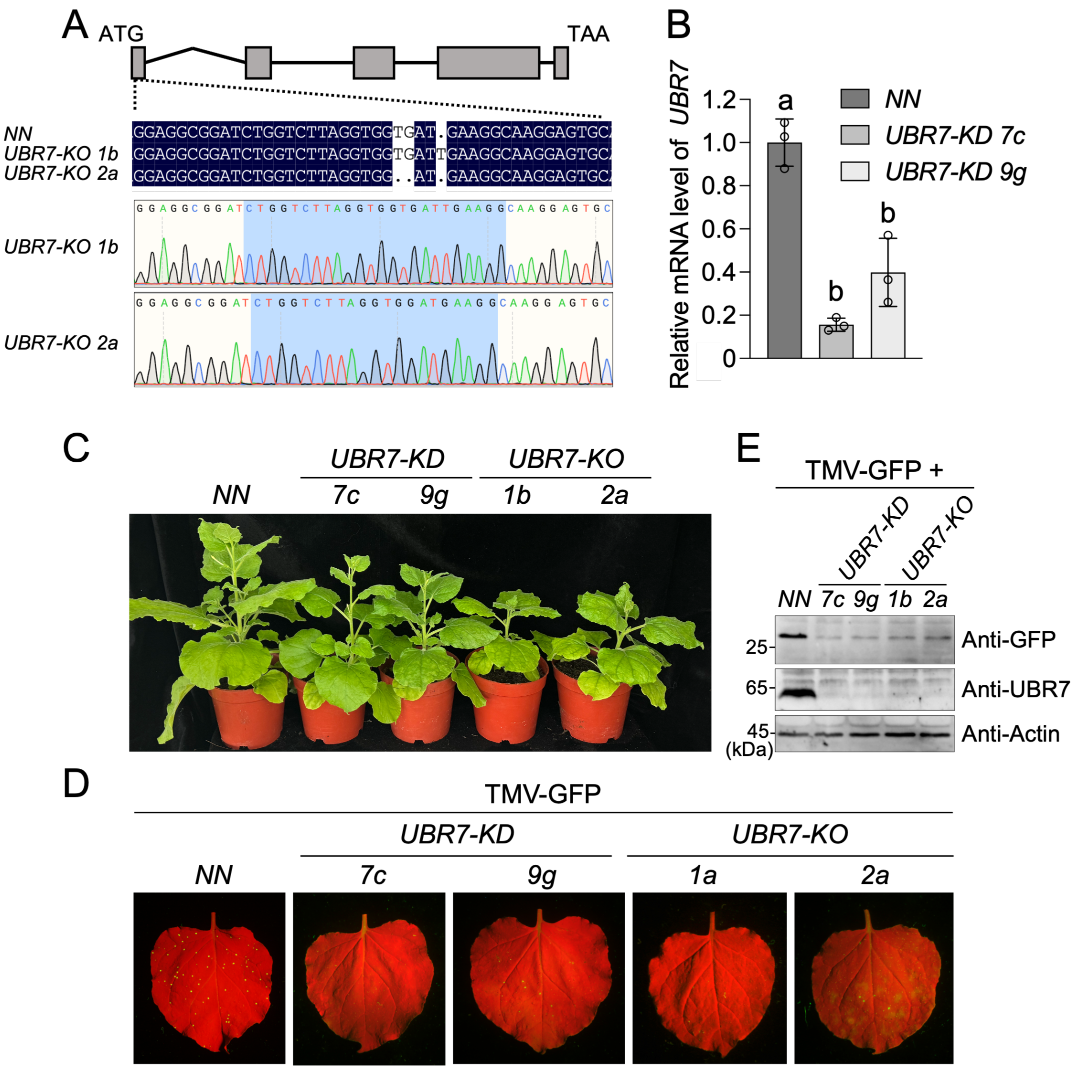

Fig. S7. Generation of *UBR7-KO* and *UBR7-KD N. benthamiana* plants in the *NN* background. (**A**) Location of the single guide RNA target within the genomic fragment of UBR7. Gray boxes represent exons, while black lines denote introns. The guide RNA was designed based on the DNA sequence of the first exon. Base insertions and deletions were observed in two independent *UBR7-KO* lines among T1 transformants. (**B**) qRT-PCR assay confirming the down-regulation of *UBR7* mRNA in the *UBR7-KD* lines. (**C**) Phenotypes of *NN*, *UBR7-KO*, and *UBR7-KD N. benthamiana* plants eight weeks after sowing. (**D**) Analysis of *N*-mediated resistance to TMV in *UBR7-KD* and *UBR7-KO N. benthamiana* plants. Leaves were mechanically inoculated with GFP-tagged TMV-U1 virions, and photographs were taken under ultraviolet light 3.5 days post-inoculation. (**E**) Immunoblot analysis of TMV-U1-GFP accumulation in the inoculated leaves, as shown in panel D. Actin served as the loading control.

A

Servers	Site	Link
GPS-uber	K20	https://gpsuber.biocuckoo.cn/online.php
RUBI 1.0	K20, K28, K36	http://old.protein.bio.unipd.it/rubi/

B

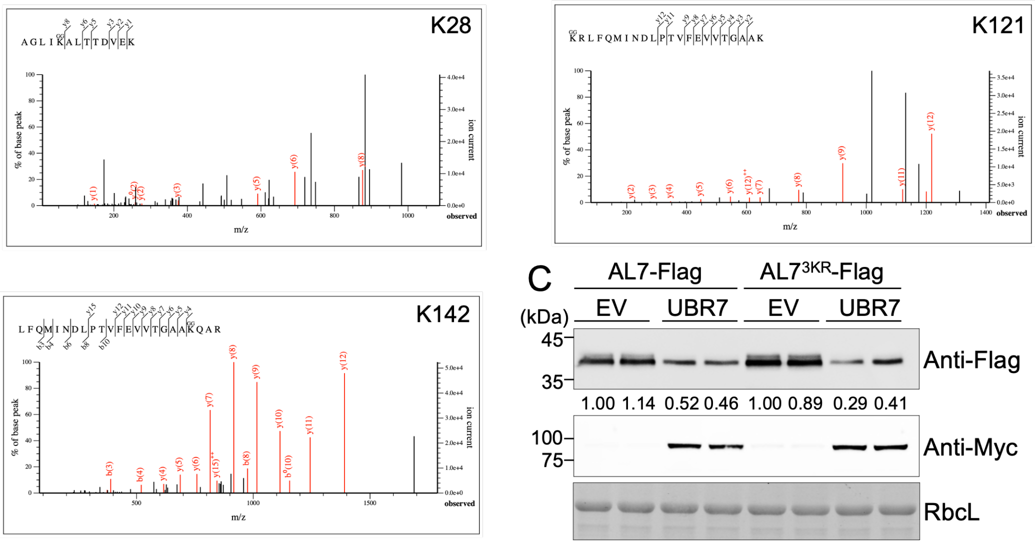

Fig. S8. Identification of ubiquitination sites within the AL7 protein. (**A**) Prediction of AL7 ubiquitination sites using various online servers. (**B**) MS spectra of the identified peptides containing Lys28, Lys121, and Lys142. The "GG" above the lysine (K) indicates potential ubiquitination at these sites. (**C**) Mutating Lys28, Lys121, and Lys142 does not affect UBR7-mediated AL7 degradation.

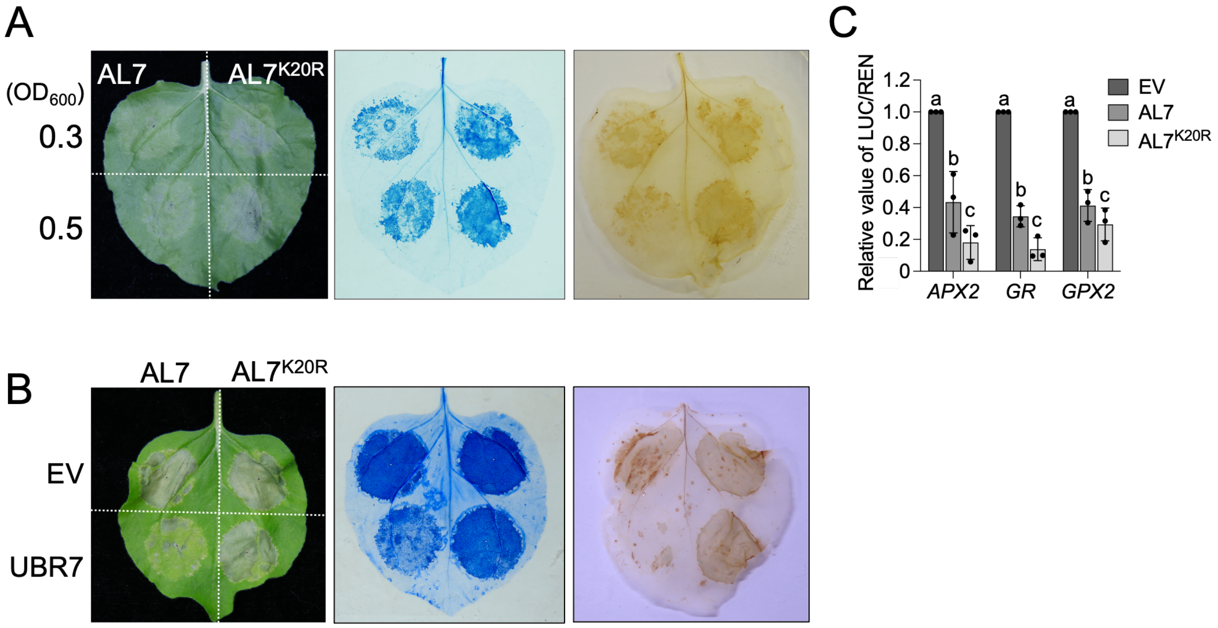

Fig. S9. UBR7 inhibits AL7-induced cell death and ROS production. (**A**) The AL7^{K20R} mutant exhibits increased cell death and ROS accumulation compared to the wild-type AL7. Photographs were taken at 2 dpi. (**B**) UBR7 does not significantly affect AL7^{K20R}-induced cell death and ROS accumulation. The experiment was conducted three times with consistent results. Photographs were taken at 4 dpi. (**C**) The AL7^{K20R} mutant demonstrates greater transcriptional repression of the *APX2*, *GR*, and *GPX2* genes.

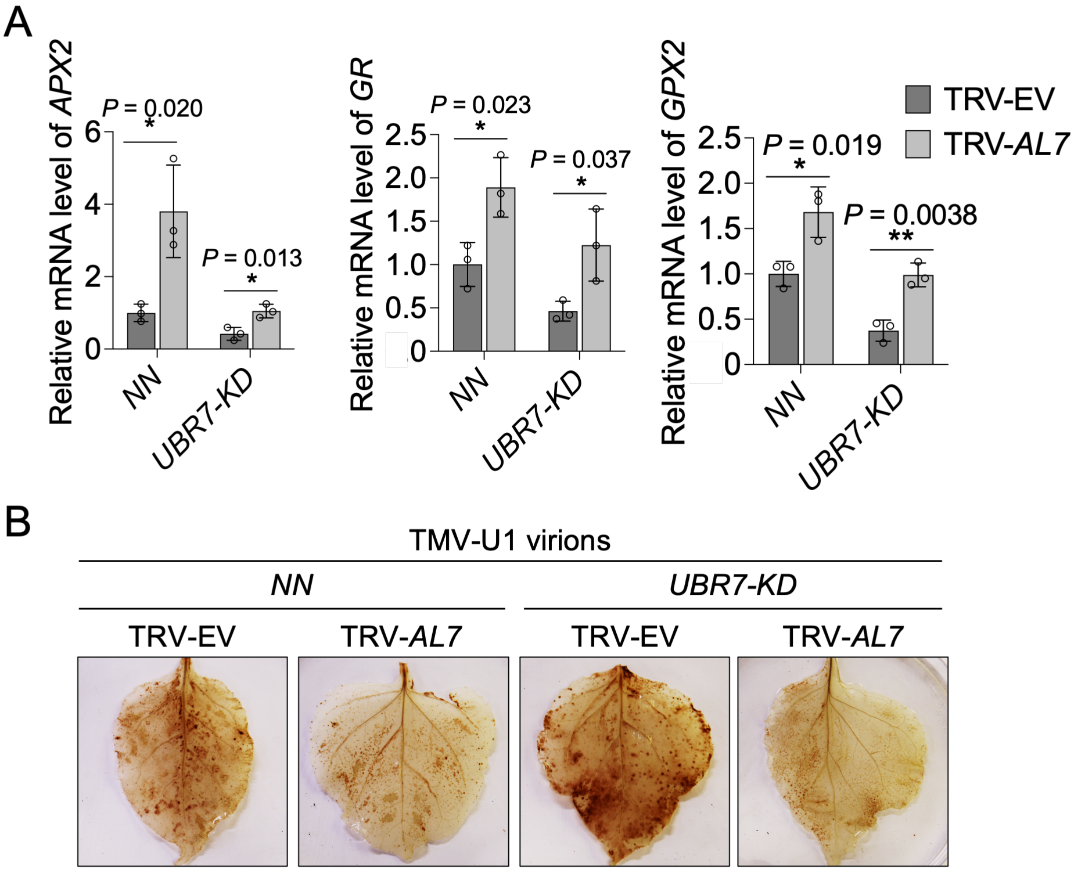

Fig. S10. UBR7 functions upstream of AL7 in regulating ROS accumulation during *N*-mediated immune responses. (**A**) qRT-PCR analysis of *APX2*, *GR*, and *GPX2* expression in TRV-EV- or TRV-*AL7*-infected *NN* and *UBR7-KD* plants. (**B**) DAB staining demonstrates that *AL7* silencing reduces ROS accumulation in *UBR7-KD* plants. 300 ng of TMV-U1 virions were rub-inoculated onto TRV-EV- or TRV-*AL7*-infected *NN* and *UBR7-KD* plants. At 48 hours after inoculation, the harvested leaves were subjected to DAB staining.

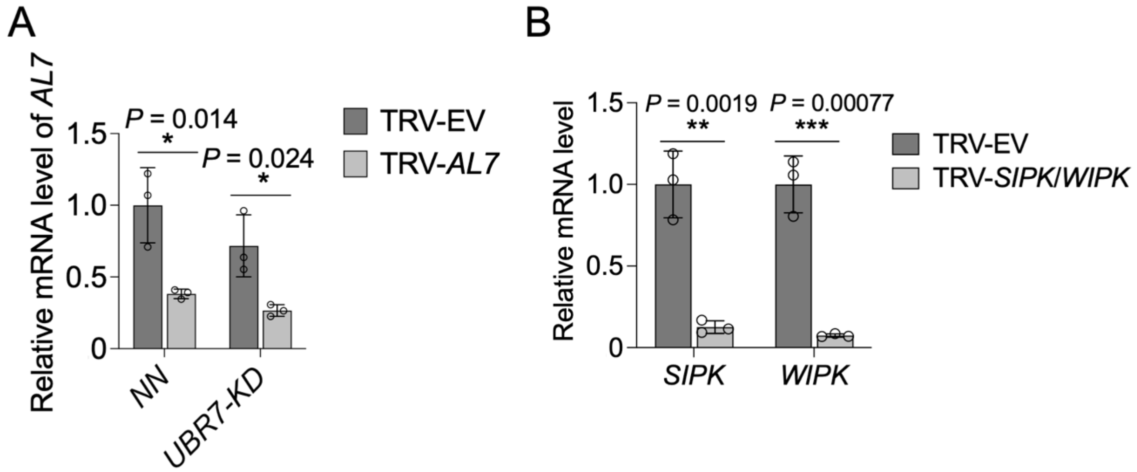

Fig. S11. qRT-PCR assay confirming the downregulation of *AL7* (A), *SIPK*, and *WIPK* (B) via the TRV-VIGS system. Error bars represent the mean ± SD (n = 3 biological replicates). Asterisks indicate significant differences determined by an unpaired two-tailed *t*-test (*$P < 0.05$; **$P < 0.01$; ***$P < 0.001$).

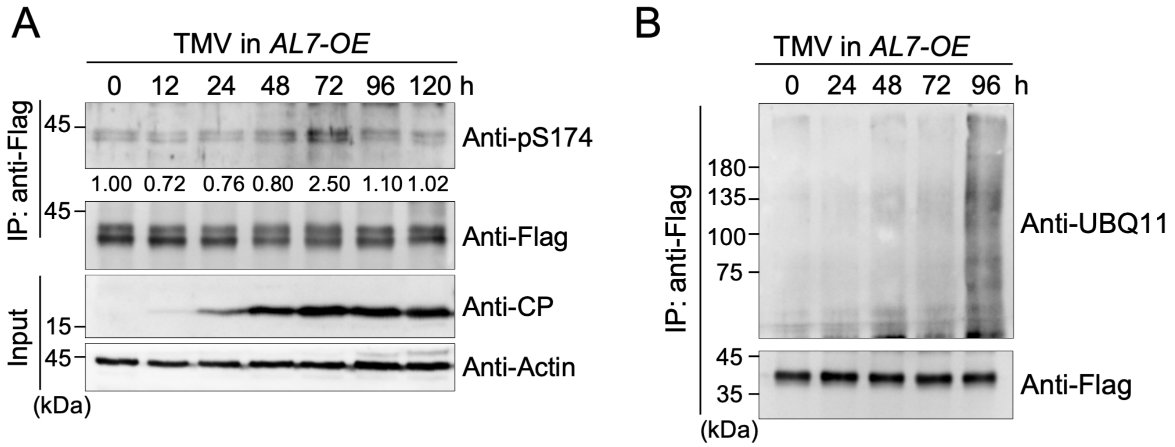

Fig. S12. Time-course analysis of AL7 phosphorylation and ubiquitination during *N*-mediated resistance against TMV. (**A**) Analysis of AL7 phosphorylation at the Ser174 site during the *N*-mediated immune response. (**B**) Analysis of AL7 ubiquitination during the *N*-mediated immune response. For (A) and (B), leaves from *AL7-OE* plants inoculated with 300 ng TMV-U1 virions were harvested at the specified time points. Total proteins were immunoprecipitated using anti-Flag beads and analyzed by immunoblotting with the antibodies indicated on the right.

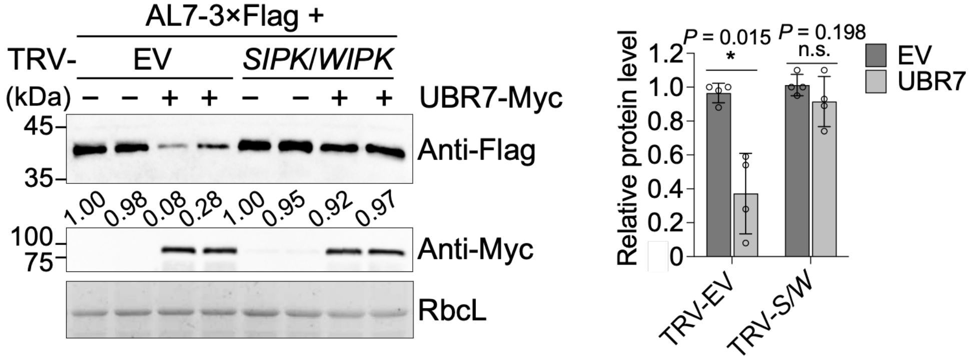

Fig. S13. SIPK and WIPK are essential for the UBR7-mediated degradation of AL7. *Agrobacterium* mixtures expressing AL7 and either EV or UBR7-Myc were infiltrated into the upper, uninoculated leaves of *N. benthamiana* plants, which were treated with TRV-EV or TRV-*SIPK/WIPK*. The infiltrated leaf tissues were sampled at 48 hours post-infiltration (hpi) and analyzed through immunoblotting. RbcL served as the loading control. A quantitative analysis of the AL7 protein levels is presented on the right. Error bars indicate the mean ± SD (n = 4 biological repeats). Asterisks above the corresponding bar chart represent significant differences determined by an unpaired two-tailed *t*-test (n.s. = not significant, $P > 0.05$; *$P < 0.05$).

Table S1. Primers used in this study.

Primer	Primer sequence (5'-3')	Purpose
207-UBR7-F	GGGGACAAGTTTGTACAAAAAAGCAGGCTCCATGGCTGATGCATTTGAAGAGG	pDONOR207-UBR7
207-UBR7-R	GGGGACCACTTTGTACAAGAAAGCTGGGTAAGTCGTACGCCTGCGCTTCTG	
CE-UBR7-F	GCCTGGCGCGCCACTAGTGATGGCTGATGCATTTGAAGAGG	pSPYCE-UBR7
CE-UBR7-R	GTCGACAGTACTATCGATGGAAGTCGTACGCCTGCGCTTCTG	
nLuc-UBR7-F	GGACGAGCTCGGTACCCGGGATCCATGGCTGATGCATTTGAAGAGGAAGG	pCAMBIA1300-NLUC-UBR7
nLuc-UBR7-R	CCCGGGACGCGTACGAGATCTGGTCGACAGTCGTACGCCTGCGCTTCTGGGC	
AL7-cLuc-F	CGTCCCGGGGCGGTACCCGGGATCCCATGGAAGGAATTCCACATCCAATACCG	pCAMBIA1300-cLUC-AL7/AL7^{S174A}/AL7^{S174D}
AL7-cLuc-R	TACGAACGAAAGCTCTGCAGGTCGACCTAAACTCTGGCCCTCTTGTTACTGC	
MBP-UBR7-F	GGATTTCAGAATTCGGATCCATGGCTGATGCATTTGAAGAGG	pMAL-C2X-UBR7
MBP-UBR7-R	CGACGGCCAGTGCCAAGCTTTAAGTCGTACGCCTGCGCTTC	
UBR7-His-F	TAAGAAGGAGATATACATAATGGCTGATGCATTTGAAGAGG	pET30a-UBR7
UBR7-His-R	GGTGGTGGTGGTGCTCGAGAGTCGTACGCCTGCGCTTCTG	
AL7-His-F	TAAGAAGGAGATATACATAATGGAAGGAATTCCACATCCAATACCG	pET30a-AL7/AL7^{S174A}/AL7^{S174D}
AL7-His -R	GGTGGTGGTGGTGCTCGAGAACTCTGGCCCTCTTGTTACTGC	
GST-AL7-F	ATCTGGTTCCGCGTGGATCCATGGAAGGAATTCCACATCCAATACCG	pGEX-KG-AL7
GST-AL7-R	CAGTCACGATGAATTAAGCTCTAAACTCTGGCCCTCTTGTTACTGC	
GST-PAL-F	ATCTGGTTCCGCGTGGATCCATGCCGAGAACCGTAGAGGAAGTTTTC	pGEX-KG-PAL
GST-PAL-R	CAGTCACGATGAATTAAGCTTCATGCCTGTTTAGCAGCTCCAGTCAC	
GST-VM-F	ATCTGGTTCCGCGTGGATCCATGAGGGATGCTGCTCATAATAACAG	pGEX-KG-VM
GST-VM-R	CAGTCACGATGAATTAAGCTTCAGAGGGTTGCCCCTTGTTCATCTTC	
GST-PHD-F	ATCTGGTTCCGCGTGGATCCATGTGTGGAGCTTGCGGTGATAATTAT	pGEX-KG-PHD
GST-PHD-R	CAGTCACGATGAATTAAGCTTCAGCAACTAGGACACTTATACTGC	
dsUBR7-F1	AGGGCGAATTGGGTACCCGAAGGACAAGGAGGTGGTAGAAG	pMDC32-dsUBR7
dsUBR7-R1	GCATGCGATACCGTCGACTACACCTACAGAGAATTTCTCTCC	
dsUBR7-F2	GGAACAAAAGCTGGAGCTCCGAAGGACAAGGAGGTGGTAGAAG	
dsUBR7-R2	AAGTGCATACTTAACTGCAGTACACCTACAGAGAATTTCTCTCC	
UBR7-gRNA-F	TGATTGTTAGGTGGTGATGAAGGCA	BGK01-UBR7
UBR7-gRNA-R	AAACTGCCTTCATCACCACCTAACA	
DEX-p50-F	TAGTCGACTCTAGCCTCGAGATGGAGATAGAGTCTTTAGAGCAGTTTC	pTA7001-p50-Flag
DEX-p50-R	GGGAGGCCTGGATCGACTAGTGAGCTCTCCGGTTGACCCTTATTTATC	
K20R-F	CAATGATTTCAGAGGCCGTAGAGCTGG	pMDC32-AL7^{K20R}-Flag

K20R-R	CTACGGCCTCTGAAATCATTGAAAACTT	
K28R-F	GGTTTAATCCGAGCACTTACCACAG	pMDC32-AL7^{3KR}-Flag
K28R-R	AGTGCTCGGATTAAACCAGCTC	
K121R-F	GTGAAAGGCGGAGGCTTTTCCAGATG	
K121R-R	AAAGCCTCCGCCTTTCACTCTTACC	
K142R-F	TGGAGCTGCTCGACAGGCAAGGGATG	
K142R-R	TGCCTGTCGAGCAGCTCCAGTCAC	
AL7-UBA1-F	AGGGAAGGATTTCAGAATTCATGGAAGGAATTCCACATCCAATACCG	pCDFDuet-MBP-AL7-UBA1-S
AL7-UBA1-R	ACGTCGTATGGGTAAGGCCTAACTCTGGCCCTCTTGTTAC	
UBR7-UBC8-F	CATCACCAAGCCAGGGATCCATGGCTGATGCATTTGAAGAGG	pACYCDuet-UBR7-MYC-UBC8-S
UBR7-UBC8-R	ATGAGCTTCTGCTCAGGCCTAGTCGTACGCCTGCGCTTC	
qSIPK-F	ACGAGCCCATTTGCATGACTCCC	qPCR
qSIPK-R	AGCTCCTTCATCTGTTCCTCCGT	
qWIPK-F	CCGATCTGCCCCGTTCCATCC	
qWIPK-R	TCAGGATTCAGCGACAAAGCTTCC	
qUBR7-F	CTGATGCATTTGAAGAGGAAGGTG	
qUBR7-R	CTTTACTATAGGTGCACTCCTTGC	
qAL7-F2	CTTGTGTCTCTATGGGCTC	
qAL7-R2	AACCAGTCCTTCTCTTGC	
qAPX2-F	ACAGAGCCACCAGTTGAAGG	
qAPX2-F	TCCCAAGGTATGTCCACCAG	
qGR-F	GGGGATAATGCAGCACCACT	
qGR-F	GTGTCCTGGGTGCATATCGT	
qGPX2-F	AAGACAAGCAAAGTTCTGGG	
qGPX2-F	CACAGTGGCATCAAAGTCC	

Data S1. List of AL7-proximal proteins.

Data S2. Peptide quantification of AL7-enriched proteins in AL7 proxitome and Citrine proxitome.

Data S3. Overlap and differences between AL7 proxitome in the absence and presence of p50.

Data S4. Overlap and differences between AL7 proxitome and AtAL7 AP-MS results.

Data S5. Subcellular localization prediction of AL7-proximal proteins.

Data S6. Kyoto Encyclopedia of Genes and Genomes (KEGG) analysis of AL7-proximal proteins.

Data S7. List of UBR7-proximal proteins.

Data S8. Subcellular localization prediction of UBR7-proximal proteins.

Data S9. KEGG analysis of UBR7-proximal proteins.
